# Screening durum wheat cultivars for resistance traits against the stem-base pathogen* Fusarium graminearum*

**DOI:** 10.7717/peerj.20105

**Published:** 2025-11-13

**Authors:** Edyta Kwiatkowska, Urszula Wachowska, Weronika Giedrojć, Agata Wachowska, Dariusz Gontarz

**Affiliations:** 1 Department of Entomology, Phytopathology and Molecular Diagnostics, University of Warmia and Mazury in Olsztyn, Olsztyn, Poland; 2 Polish Cereal Plant, Lubella, Lublin, Poland

**Keywords:** Fusarium crown rot, *Fusarium graminearum*, *F. culmorum*, Chemical and biological seed dressing

## Abstract

Durum wheat is not a traditional crop in countries with a temperate climate, but the growing demand for semolina in the food processing industry has increased the popularity of this cereal species in the farming sector. The pathogens responsible for Fusarium crown rot (FCR), eyespot, and sharp eyespot contribute to lodging, disrupt the translocation of water and nutrients in plants, and decrease yields. The present study was conducted in several dozen locations in Poland to determine the severity of FCR, eyespot, and sharp eyespot in more than ten durum wheat cultivars, to identify stem-base pathogens, to analyze the virulence of *Fusarium graminearum*, and to evaluate the effectiveness of seed dressing in reducing the severity of infections in durum wheat seedlings. Durum wheat cultivars were screened in field and plot experiments in Poland. The virulence of *F. graminearum* and the effectiveness of biological and chemical seed dressing in reducing the incidence of FCR were determined *in vivo*. In the studied locations, symptoms of FCR and eyespot were observed on 16–76% and 1–36% of durum wheat stems, respectively, on average. Sharp eyespot was noted only in one year of the study in a single location (1–6%). The severity of the analyzed diseases was generally low. *Fusarium avenaceum*, *F. culmorum*, *F. graminearum*, *F. oxysporum*, *F. poae*, *F. solani*, and *F. sporotrichioides* were isolated from stems with symptoms of FCR. The number of *F*. *graminearum* and *F. culmorum* isolates was significantly positively correlated with the severity of FCR symptoms (*r* = 0.480 and *r* = 0.485, respectively). *Fusarium graminearum* was identified in 15 locations, and *F. culmorum* was detected in six locations. Seed treatment with triticonazole reduced the number of ungerminated kernels by 42.6% in comparison with the untreated control. Seed treatment involving *Debaryomyces hansenii* decreased the number of ungerminated kernels by 31.1% on average, but this effect was noted only in cv. Floradur. All durum wheat cultivars evaluated in Koch’s postulate test were severely infected, but *F. graminearum* isolates differed in virulence. *Fusarium graminearum* was the most prevalent pathogen in durum wheat stands, and it was highly virulent for seedlings. To decrease the incidence of FCR in durum wheat stands, new resistant varieties should be tested, suitable farming locations with desirable soil and environmental conditions should be identified, and new agricultural treatments should be developed.

## Introduction

Durum wheat (*Triticum turgidum* L. subsp. *durum* Desf.) occupies 7–8% of the total land area under all wheat species (Martínez-Moreno, Ammar & Solís, 2022). More than half of the total land area under durum wheat is located in the Mediterranean Region, including in Southern Europe (Visioli et al., 2022). Durum wheat was domesticated around 10,000–15,000 BCE in the Mediterranean Region, and it is currently used in the production of pasta, couscous, bulgur, puddings, pastries, freekeh, kishk, and other traditional dishes (Ficco et al., 2014). Durum wheat is not a traditional crop in Poland, but changing consumer preferences and the growing demand for semolina in pasta production have increased the popularity of this crop species in countries with a temperate climate (Bobryk-Mamczarz, Kiełtyka-Dadasiewicz & Rachoń, 2021; Banach, Majewska & Zuk Gołaszewska, 2021). In Poland, only four winter cultivars of durum wheat (Ceres, SM Eris, SM Metis, and SM Tetyda) have been placed on the Polish National List of Agricultural Plant Varieties (Central Research Centre for Cultivated Plant Varieties, 2022), and most cultivars that are currently grown in Poland have been developed in Slovakia, Germany, and Austria. Polyphagous pathogens that infect durum wheat leaves, spikes, and stems, including common wheat pathogens, pose a significant threat to durum wheat cultivation (Wachowska et al., 2023).

*Fusarium* root rot (FRR), Fusarium crown rot (FCR) (*Fusarium* spp.), eyespot (*Oculimacula* spp.), and sharp eyespot (*Rhizoctonia cerealis*) are *stem*-*base* diseases of cereals (Lemańczyk & Kwaśna, 2013; Kuzdraliński et al., 2014). Species of the genera *Fusarium* and *Rhizoctonia* infect the stem base and the roots of wheat plants, whereas species of the genus *Oculimacula* colonize only the stem base (Ray et al., 2006; Lemańczyk & Kwaśna, 2013; Lisiecki et al., 2022; Yang et al., 2022). *Fusarium* pathogens also infect leaves (Fusarium leaf blight, FLB) and spikes (Fusarium head blight, FHB) and produce numerous mycotoxins that cause human and animal diseases known as mycotoxicoses (Ji et al., 2019). *Fusarium* root rot is one of the most important cereal diseases worldwide which compromises water uptake by plants and poses a particular threat during prolonged drought (Beccari, Covarelli & Nicholson, 2011; Bouarda et al., 2022). In most cases, the pathogens responsible for FCR do not infect roots, and the main symptoms of infection include brown patches or black scars between the stem base and the first and second internode (Paulitz, 2006). The severity of FRR and FCR increases during the ripening stage, which leads to lodging, chalky appearance of spikes, disruptions in the translocation of water and nutrients in plants, decrease in yields, and lower grain quality (Bouarda et al., 2022). In wheat, these diseases are caused by several *Fusarium* species, mainly *F*. *culmorum*, *F*. *graminearum* (sexual stage: *Gibberella zeae*), and *F*. *pseudograminearum* (sexual stage: *G*. *coronicola*) (Kazan & Gardiner, 2018; Dyer et al., 2009; Deng et al., 2018; Bouarda et al., 2022). *Fusarium graminearum* and *F*. *pseudograminearum* cause both FHB and FCR. *Fusarium graminearum* is isolated mainly from spikes, whereas *F*. *pseudograminearum* infects the stem base. The prevalence of these pathogens differs across agricultural regions (Akinsanmi et al., 2006).

The infectious process caused by *Fusarium* pathogens in the stem base begins when fungal hyphae penetrate the sheathing leaf base that encircles the stem. In plants inoculated with *F*. *culmorum*, stem tissues were colonized extensively by the pathogen, but the spikes were affected only in the ripening stage (Covarellia et al., 2012). Other studies have also shown that *Fusarium* species are unable to spread from the stem base to spikes (Gelisse et al., 2006). However, toxins can be translocated from roots to spikes (Covarellia et al., 2012; Winter et al., 2019; Bouarda et al., 2022). According to Wang et al. (2018), *F*. *graminearum* is a virulent pathogen of wheat roots that forms highly specialized infection structures. Durum wheat is highly susceptible to *F*. *culmorum* and *F*. *graminearum* infections (Dodman & Wildermuth, 1987; Dyer et al., 2009; Bouarda et al., 2022; Wachowska et al., 2023).

In Poland, the severity of stem-base infections remains unknown because the area under durum wheat is relatively small. In addition, soil-borne *Fusarium* species that cause FRR and FCR are less frequently studied than FHB pathogens which have been researched extensively in the last 30 years. The toxin-producing *F. graminearum* species (Wachowska et al., 2023) poses a significant threat to durum wheat spikes (Wachowska et al., 2023), and its pathogenicity for durum wheat seedlings and stem bases was evaluated in this study. The present study was conducted in 2018–2019 to determine the severity of FCR (*Fusarium* spp.), eyespot (*Oculimacula* spp.), and sharp eyespot (*Rhizoctonia* spp.) in durum wheat cultivars, to identify stem-base pathogens, to analyze the virulence of *F. graminearum* colonizing durum wheat seedlings, and to assess the effectiveness of biological and chemical seed dressing in reducing the severity of infections in durum wheat seedlings.

## Materials and Methods

### Stem sampling in field and plot experiments

**Figure 1:**
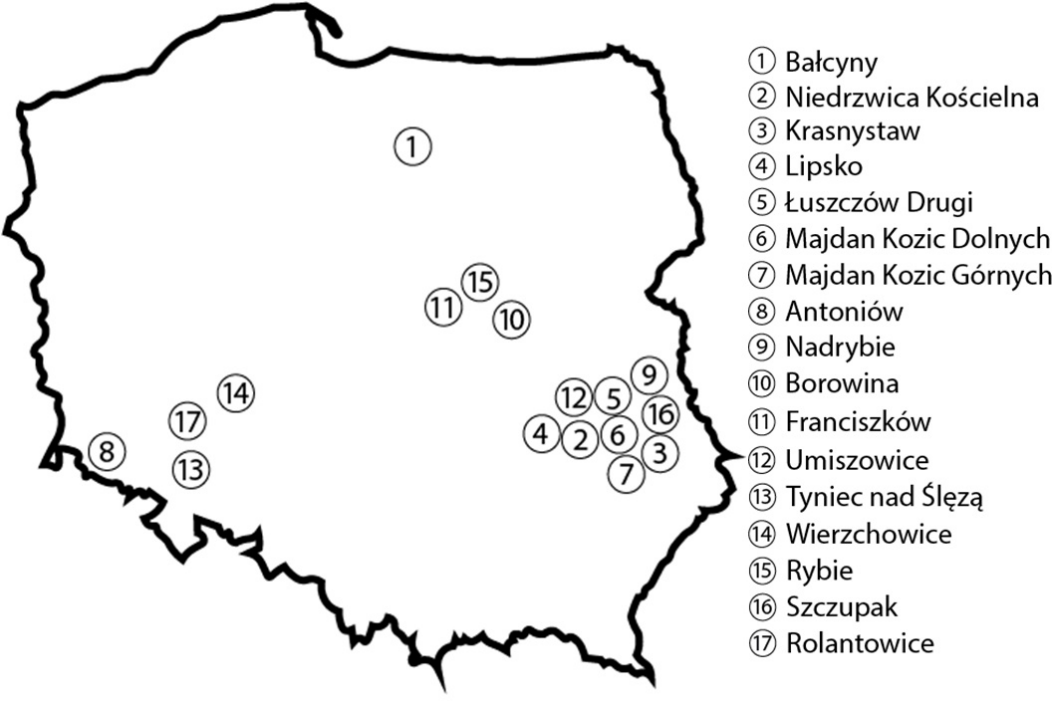
Location of the analyzed experimental plots and agricultural fields on the map of Poland. Created using Adobe Illustrator.

Field experiments were conducted in 24 agricultural fields, 16 of which were located in southern Poland (Fig. 1). The analyzed fields were separated by a distance of minimum three km. Each field had an area of 2,000–10,000 m^2^. Samples of durum wheat stems were collected randomly from five locations in each field in a zig-zag pattern. Each sampling site had an area of 1–2 m^2^, and sampling sites were separated by a distance of 10–30 m. Ten plants were sampled from each site, and a total of 50 plants were collected from each field. Durum wheat stems were sampled in July 2018 in the ripening stage (BBCH 83-92) (Agriculture and Horticulture Development Board (AHDB), 2018). The fields were cultivated by local farmers in 16 locations, and all farmers were supplied with durum wheat seeds by a commercial pasta producer (Lubella Food Ltd. LP in Lublin, Poland; Suchowilska et al., 2022). Five spring cultivars (Duragold, Durasol, Duranegra, Floradur, Tamadur, vegetation from March to July 2018) and seven winter cultivars (Auradur, Elsadur, Karmadur, Pentadur, Spiradur, Tempodur, Wintergold, vegetation from September 2017 to July 2018) of durum wheat were grown in the studied fields. A total of 1,200 durum wheat stems were evaluated for symptoms of infection, and severely infected stems were selected for mycological analyses. A permit to conduct the field trails was not required. All the fields were sown with different wheat durum varieties in the confines of contract with company produced pasta.

In small-area experiments, established in north-eastern (Bałcyny, location 1) and south-eastern (Niedrzwica Kościelna, location 2) Poland, durum wheat stems were sampled in July 2018 and in July 2019 (Fig. 1). The experimental plots had an area of 6 m^2^ each. The experimental factors were year (factor 1), location (factor 2), and cultivar (factor 3). Five spring cultivars of durum wheat (Durasol, Duranegra, Duragold, Floradur, and Tamadur) were sown in experimental plots. The experiment had a randomized block design with four replicates. Detailed information about the agricultural treatments applied in each location is presented in Table 1. Twenty-five stems were sampled from each plot in two growing seasons, and a total of 1,000 stems (100 stems per treatment) were evaluated for symptoms of infection.

**Table 1:** Chemical treatments applied in field experiments involving *Triticum turgidum* ssp. *durum*.

Treatment	Commercial name	Content of active ingredient	Dose	Application date (BBCH stage)
Growth regulator	Moddus 250 EC	Trinexapac ethyl (cyclohexanedione derivative class) - 250 g/L (25.5%)	Two applications at 0.28 L/ha each	BBCH 31, BBCH 41
Herbicide	Legato 500 SC	Diflufenican (phenoxynicotine anilide herbicide) - 500 g/L	0.3 L/ha	BBCH 12–13
Fungicide	Wirtuoz 520 EC	Prochloraz (imidazole herbicide) - 320 g/L (28.53%), tebuconazole (triazole herbicide) - 160 g/L (14.27%), proquinazid (quinazolinone herbicide) - 40 g/L (3.57%)	1 L/ha	BBCH 31
	Priaxor^®^ + Bumper 250 EC	Pyraclostrobin (strobilurin fungicide) - 150 g/L (14.63%), fluxapyroxad (carboxamide fungicide) - 75 g/L (7.32%).+ propiconazole (triazole fungicide) - 250 g/L (25.10%).	0.7 L/ha + 0.5 L/ha	BBCH 54

### Assessment of stem health

The leaf sheath was removed from the stem base, and stems were subjected to a phytopathological analysis. The percentage of plants with symptoms of FCR, eyespot, and sharp eyespot was determined. The severity of disease symptoms was assessed on a four-point scale (0—healthy stem, 1—weakly infected stem with one patch covering up to 25% of stem area, 2—strongly infected stem with several patches covering 25–50% of stem area, 3—rotting stem); European and Mediterranean Pllat Protection Organization (EPPO), 2006. Severely infected stems were selected for mycological analyses.

### Pathogen isolation and identification by the culture-based method

Infected stems collected from each field/plot were carefully washed under running water. A total of 264 stems with symptoms of FCR (six stems from each sampling site in field experiments or from each treatment in plot experiments), 20 stems with symptoms of eyespot (from field and plot experiments), and 10 stems with symptoms of sharp eyespot (from plot experiments) were examined in the mycological analysis. Stem fragments were surface disinfected by immersion in 75% ethanol (Stanlab, Lublin, Poland) for 1 min and 2% sodium hypochlorite (Stanlab) for 2 min. Disinfected stems were rinsed with sterile water three times and dried on blotting paper (Eurochem BGD, Tarnów, Poland). Stem fragments with a length of 3–5 mm were placed on potato dextrose agar (PDA, A&A Biotechnology, Poland) containing 150 µg/mL of streptomycin (Serva, Heidelberg: Germany) and 75 µg/mL of kanamycin (A&A Biotechnology, Gdansk: Poland). Petri plates (FLMedical, Torreglia, Italy) were incubated in dark at a temperature of 24 °C for 7 days (Pol-Eko incubator; Pol Eko, Wodzislaw Slaski, Poland). The emerged colonies were transferred to fresh PDA, and *Fusarium* spp. were identified to species level on synthetic low-nutrient agar (SNA containing one g of KH_2_PO_4_, one g of KNO_3_, 0.5 g of KCl, 0.5 g of MgSO_4_ ×7 H_2_O, 0.2 g of glucose, 0.2 g of sucrose, 20 g of agar, up to one L of distilled water, with the addition of streptomycin (Serva) and kanamycin (A&A Biotechnology; Leslie & Summerell, 2006). Fungal species were identified with the use of keys based on the morphological structure of mycelia, hyphae, and conidia (Gonzalez Garcia, Portal Onco & Rubio Susan, 2006; Leslie & Summerell, 2006; Sharon et al., 2006; Gargouri et al., 2022).

### DNA isolation and molecular identification of *F. graminearum*

*Fusarium graminearum* isolates selected for the pathogenicity test were identified in the polymerase chain reaction (PCR) assay. DNA was isolated with the Bread-Beat Micro AX Gravity kit (A&A Biotechnology) according to the manufacturer’s instructions. The reaction mix consisted of 2x PCR taqNOVA-RED master mix (BLIRT, Gdansk, Poland) and 20 ng of the isolated DNA. The partial sequence of the small subunit 18S rDNA gene, 5.8S rDNA region, the partial sequence of the large subunit 28S rDNA gene and conserved non-coding regions (ITS) were amplified with the use of primers specific to ITS4 and ITS5 (ITS5 (F) GTATCGGACGGAGATCCAGC, ITS4 (R) TTGCTCAGTGCATTGTCGG). The amplification reaction was conducted in the Mastercycler Ep Gradient thermal cycler (Eppendorf, Germany). The reaction had the following thermal profile: initial denaturation at 95 °C (3 min), followed by 34 cycles of: denaturation at 95 °C (1 min), hybridization at 58 °C (1 min), elongation at 74 °C (3 min), and extension at 74 °C (10 min) (Winter et al., 2019). The size of ITS products was determined by electrophoresis on 1% agarose gel (Prona AG) in the presence of ethidium bromide in TBE buffer (BLIRT). The products separated on agarose gel were visualized with a transilluminator (UVP). Amplification products were sequenced by Genomed S.A. in Warsaw (Genomed, 2020, Poland). Fungal isolates were identified by comparing sequences with the BLAST tool in the NCBI database (NCBI, 2017).

### Standard seed germination test

Seed germination tests (International Seed Testing Association (ISTA), 2019) were conducted with the use of a modified blotter test. Durum wheat cultivar (Durasol, Duranegra, Duragold, Floradur, and Tamadur) and seed dressing (chemical and biological) were the experimental factors. Chemical seed dressing involved the Triter 050 FS fungicide (active ingredient: triticonazole (triazole fungicide) –50 g/L (4.9%) which was applied according to the manufacturer’s instructions. Biological seed dressing involved a suspension of *Debaryomyces hansenii* (GenBank accession number KX444669) which was prepared according to a previously described method (Wachowska et al., 2023). Suspension density was determined at 10^6^ cells/one mL of sterile water in the Thoma cell counting chamber (Marienfeld, Lauda-Königshofen, Germany). Grain was dressed by immersion in the *D. hansenii* suspension or the fungicide for 40 min. Twenty dressed kernels of each durum wheat cultivar (harvested in 2020) were placed between four layers of moist blotter paper (Chemland, Stargard, Poland), and the paper was rolled. The rolls were placed in glass containers and incubated at a temperature of 24 °C in a phytotron (Hakman/ PHU Chłodnictwo). Kernels immersed in sterile water were the control. The experiment was conducted in four replicates. After 21 days of incubation, seedling height and root length were measured, the Normalized Difference Vegetation Index (NDVI) was calculated, and the number of seeds that produced healthy seedlings, the number of ungerminated seeds, and the number of infected seedlings were determined. The severity of seedling infection was evaluated on a four-point scale, where: 0—healthy seedlings, 1—single brown patches on the coleoptile, 2—coleoptile penetrated by *F*. *graminearum* hyphae, 3—ungerminated grain completely colonized by *F*. *graminearum* hyphae.

### Koch’s postulate test

The pathogenicity of *F. graminearum* isolates for the seedlings of several durum wheat cultivars was determined in Koch’s postulate test under controlled conditions. Five *F. graminearum* isolates—F2 (NCBI accession number MZ827460), F3 (MZ827461), F12 (MZ827462), F14 (MZ827463), and Fg3 (MZ820057)—were incubated on PDA in dark at a temperature of 25 °C for 7 days. Twelve PDA discs (with a diameter of five mm) overgrown by the hyphae of the cultured fungal isolates were placed inside a Petri plate with a diameter of nine cm (FLMedical) on moist blotter paper (Eurochem BGD). Surface-disinfected grain of durum wheat cvs. Duragold, Durasol, Duranegra, Tamadur, and Floradur was placed on PDA discs. Grain was surface disinfected by immersion in 75% ethanol (Stanlab) for 1 min and 1% sodium hypochlorite solution (Chempur, Poland) for 2 min. Disinfected grain was rinsed with sterile water three times and dried on blotter paper (Eurochem BGD). PDA discs without the pathogen were the control. The test was conducted in four replicates. Grain was germinated in a phytotron (Hakman/PHU Chłodnictwo) with a 12 h photoperiod (12 h of light/12 h of darkness) at a temperature of 23/19 °C and relative humidity of 60/80% (±5%) for 21 days. The severity of seedling infection was assessed on a four-point scale, where: 0—healthy seedlings, 1—single brown patches on the coleoptile, 2—coleoptile penetrated by *F*. *graminearum* hyphae, 3—ungerminated grain completely colonized by *F*. *graminearum* hyphae.

### Statistical analysis

Differences in infection severity in plot experiments (where year, cultivar, and location were the experimental factors) were determined by three-way analysis of variance (ANOVA). Differences in the pathogenicity of *F. graminearum* isolates in Koch’s test (where *F. graminearum* strains and durum wheat cultivar were the experimental factors) were determined by two-way ANOVA. All statistical analyses were conducted in Statistica v. 13.3 (StatSoft, Tulsa, OK, USA). In the standard seed germination test, the experimental factors were seed dressing (factor 1) and cultivar (factor 2). Pearson’s correlation coefficients were calculated to analyze the relationship between the severity and prevalence of stem-base diseases and the prevalence of the isolated pathogens. The prevalence of the predominant pathogen species isolated from the experimental plots was determined according to the following formula: 100× (total number of isolates of a given species obtained from durum wheat stems in an experimental plot)/ total number of stems from which pathogens were isolated. The efficacy of chemical and biological seed treatments was calculated using the modified Abbott’s formula: Efficacy = 100 × (1 − (T/UT)), where T is the mean disease severity in a given treatment and UT is the mean disease severity in the control treatment.

## Results

### Field experiments

In agricultural fields, symptoms of FCR and eyespot were observed in 16–65% and 2–36% of the examined durum wheat stems, respectively (Table 2, Table S1). The severity of the analyzed diseases was generally low, and the severity of FCR was high in only four cases, including in spring durum wheat cv. Duragold in Krasnystaw (2.3 on the four-point scale), cv. Duranegra in Nadrybie (2.1), cv. Durasol in Antoniów (2.2), and in winter durum wheat cv. Spiradur in Majdan Kozic Górnych (2.2) (Table 2). *Fusarium graminearum* was isolated from the stem base in 13 locations, *F. oxysporum* was isolated in eight locations, and *F. culmorum* in four locations (Table 3). The remaining pathogenic species were isolated from durum wheat stems sampled in three locations (*F. poae*), two locations (*F. avenaceum*, *F. solani*), and one location (*Microdochium nivale*, *F. sporotrichioides*). Although symptoms of sharp eyespot were not observed in the fields, *Rhizoctonia* sp. colonies were isolated from FCR-affected stems in four locations. *Oculimacula* sp., the causative agent of eyespot (which was frequently observed), were isolated only in four locations.

**Table 2:** Severity of stem-base diseases in the analyzed agricultural fields in 2018.

Cultivar (location)^*^	Fusarium crown rot		Eyespot	
	Prevalence	Severity	Prevalence	Severity
	Spring cultivars			
Duragold (3)	34.0^e-h^(±9.577)	2.3^a^(±0.076)	8.0^h-j^(±2.577)	1.3^c-e^(±0.029)
Duranegra (4)	27.0^h-j^(±4.577)	1.8^b-e^(±0.024)	0.0^n^(±0.000)	0.0^h^(±0.000)
Duranegra (5)	16.0^l-m^(±2.577)	0.9^h^(±0.035)	14.0^f-g^(±5.577)	0.9^f-g^(±0.035)
Duranegra (6)	41.0^d-e^(±7.577)	1.8^c-e^(±0.035)	11.7^g-h^(±0.333)	1.2^c-g^(±0.003)
Duranegra (8)	36.3^d-g^(±11.202)	1.8^c-e^(±0.032)	3.7^l-n^(±0.333)	1.0^f-g^(±0.033)
Duranegra (9)	65.0^a^(±11.577)	2.1^a-d^(±0.088)	7.7^i-k^(±0.333)	1.3^c-e^(±0.029)
Duranegra (10)	30.0^f-i^(±7.577)	1.1^f-h^(±0.030)	2.0^m-n^(±0.000)	1.0^f-g^(±0.033)
Durasol (8)	25.0^i-k^(±4.577)	2.2^a-b^(±0.068)	14.7^f-g^(±3.667)	1.4^b-c^(±0.057)
Floradur (11)	29.3^g-i^(±4.177)	1.5^e-g^(±0.086)	4.0^k-m^(±0.577)	0.9^f-g^(±0.035)
Floradur (8)	43.7^c-d^(±10.333)	1.6^e^(±0.035)	7.7^i-k^(±0.333)	1.3^c-e^(±0.028)
Tamadur (12)	15.3^m^(±1.201)	1.0^h^(±0.032)	7.3^i-l^(±0.333)	1.3^c-e^(±0.028)
Tamadur (12)	30.0^f-i^(±5.577)	1.1^g-h^(±0.088)	10.0^h-j^(±4.577)	0.9^g^(±0.057)
Tamadur (10)	61.3^a^(±10.667)	1.6^e-f^(±0.031)	36.0^a^(±3.377)	1.0^e-g^(±0.057)
	Winter cultivars			
Auradur (2)	50.7^b-c^(±12.882)	1.6^e-f^(±0.291)	30.0^bc^(±3.577)	1.4^b-c^(±0.057)
Elsadur (13)	37.0^d-f^(±4.882)	1.5^e-g^(±0.029)	19.7^e^(±2.185)	0.9^f-g^(±0.035)
Karmadur (14)	41.0^d-e^(±9.577)	1.7^d-e^(±0.055)	11.0^g-i^(±0.577)	1.3^c-d^(±0.111)
Pentadur (15)	35.0^e-g^(±8.577)	1.5^e-g^(±0.044)	10.0^h-j^(±2.577)	1.1^d-g^(±0.100)
Pentadur (16)	15.7^l-m^(±1.856)	1.1^g-h^(±0.057)	7.0^j-l^(±0.577)	1.0^e-g^(±0.057)
Spiradur (7)	53.0^b^(±0.577)	2.2^a-c^(±0.079)	1.7^m-n^(±0.333)	2.0^a^(±0.033)
Spiradur (16)	27.0^h-j^(±5.577)	1.7^e^(±0.035)	25.0^d^(±9.577)	1.6^b^(±0.055)
Spiradur (16)	23.0^i-l^(±4.577)	1.1^g-h^(±0.049)	3.7^l-n^(±0.333)	1.0^e-g^(±0.057)
Tempodur (12)	57.7^a-b^(±10.882)	1.8^b-e^(±0.067)	16.0^e-f^(±4.577)	1.4^b-d^(±0.005)
Wintergold (12)	18.7^k-m^(±3.882)	1.7^d-e^(±0.057)	24.0^d^(±3.577)	0.9^f-g^(±0.005)
Wintergold (17)	21.0^j-m^(±3.512)	1.0^h^(±0.030)	34.0^ab^(±4.154)	1.2^c-f^(±0.052)

**Notes.**

* (2-17) - The location of agricultural fields where the examined cultivars were grown is shown in Fig. 1.

**Table 3:** Pathogens isolated from the stem base of durum wheat with symptoms of FCR and eyespot in the analyzed agricultural fields.

Pathogen	Number of fields (location ID number)	Cultivar	Number of colonies	Percentage of colonies^*^
*F. avenaceum*	2 (12, 14)	Wintergold, Karmadur	8	36.36–60.00
*F. culmorum*	4 (8, 9, 13, 14)	Tamadur, Duranegra, Spiradur	21	7.14–85.71
*F. graminearum*	13 (3, 4, 5, 7, 8, 9, 10, 11, 12, 13, 15, 16, 17)	Tamadur, Duranegra, Spiradur, Karmadur, Floradur, Durasol, Duragold, Pentadur, Wintergold, Auradur)	241	5.56–100
*F. oxysporum*	8 (6, 7, 8, 9, 10, 12, 13, 16)	Auradur, Tempodur, Tamadur, Spiradur, Duranegra	24	9.09–36.36
*F. poae*	3 (4, 12, 16)	Tamadur Duranegra, Spiradur	15	27.78–100
*F. solani*	2 (12, 16)	Wintergold, Spiradur	2	5.56–20.00
*F. sporotrichioides*	1 (16)	Spiradur	7	38.89
*Microdochium nivale*	1 (6)	Spiradur	3	50.00
*Oculimacula* spp.	4 (8, 12, 13, 16)	Tempodur, Elsadur, Duragold, Duranegra, Spiradur	15	9.09–27.28
*Rhizoctonia* spp.	4 (4, 8, 12, 17)	Wintergold, Tamadur, Duranegra, Durasol	16	4.76–100

**Notes.**

* Only pathogen colonies isolated from a given location/field were considered.

### Plot experiments

**Figure 2:**
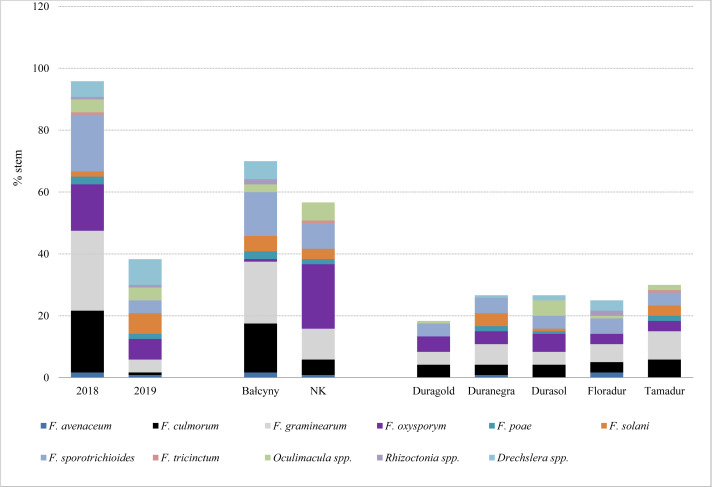
Pathogens isolated from the stem base of durum wheat with symptoms of Fusarium crown rot, eyespot, and sharp spot in the analyzed experimental plots. NK, Niedrzwica Kościelna.

In experimental plots, the prevalence of stem-base diseases was significantly higher in 2018 than in 2019 (Table 4 and Table S2). Fusarium crown rot affected most stems in both locations (31.25–75.53%), whereas symptoms of sharp eyespot were observed only in Bałcyny in all durum wheat cultivars in 2018 (Table 4). Symptoms of *Rhizoctonia* spp. infection were observed in 5.91% of Floradur stems (disease severity –1.02). Eyespot affected maximum 19.61% of the examined stems. On average, the prevalence of infections caused by *Oculimacula* spp. was significantly lower in durum wheat cvs. Duramonte and Duramant than in the remaining cultivars. Fungi *Fusarium graminearum* and *F. culmorum* were isolated from the stem base mainly in 2018 (25.8% and 20% of stems, respectively), especially in Bałcyny location (20% and 15.8% respectively) (Fig. 2). *Fusarium oxysporum* was isolated from all durum wheat cultivars grown in both locations. This pathogen was identified on 20.8% of the stems in Niedrzwica Kościelna. On average, *F. oxysporum* was most frequently isolated (5.8%) from the stem bases of durum wheat cv. Durasol (Fig. 2).

**Table 4:** Prevalence and severity of stem-base diseases in the analyzed experimental plots.

Year	Location	Cultivar	Fusarium crown rot		Eyespot		Sharp eyespot	
			Prevalence	Severity	Prevalence	Severity	Prevalence	Severity
2018	Bałcyny	Floradur	75.53^ab^ (±9.80)	1.69^a^ (±0.13)	10.56^ab^(±2.98)	1.06^bc^(±0.11)	5.95^a^ (±4.54)	1.02^a^ (±0.05)
		Duranegra	80.99^a^ (±10.43)	1.82^a^ (±0.08)	10.07^ab^(±6.79)	0.94^bc^(±0.39)	1.51^b^ (±2.07)	0.50^ab^(±0.53)
		Durasol	67.34^abc^(±9.18)	1.62^a^ (±0.16)	19.08^a^ (±8.48)	1.23^bc^(±0.18)	1.27^b^ (±1.87)	0.38^ab^(±0.52)
		Duragold	74.06^ab^(±5.21)	1.69^a^ (±0.16)	11.89^ab^(±2.68)	1,19^bc^(±0.19)	1.00^b^ (±1.07)	0.50^ab^(±0.53)
		Tamadur	66.19^abc^(±11.21)	1.76^a^ (±0.16)	19.61^a^ (±5.69)	1,10^bc^(±0.12)	2.00^ab^(±2.62)	0.50^ab^(±0.53)
	Niedrzwica Kościelna	Floradur	67.38^abc^(±9.96)	1.64^a^ (±0.28)	11.35^ab^(±1.89)	1.22^bc^(±0.21)	0^b^ (±0.00)	0^b^ (±0.00)
		Duranegra	56.40^abc^(±9.37)	1.59^a^ (±0.11)	8.20^ab^(±3.34)	1.19^bc^(±0.21)	0^b^ (±0.00)	0^b^ (±0.00)
		Durasol	63.24^abc^(±11.93)	1.58^a^ (±0.21)	13.62^ab^(±5.24)	1.05^bc^(±0.07)	0^b^ (±0.00)	0^b^ (±0.00)
		Duragold	70.58^abc^(±13.69)	1.57^a^ (±0.19)	5.61^ab^(±4.55)	1.08^bc^(±0.14)	0^b^ (±0.00)	0^b^ (±0.00)
		Tamadur	71.50^abc^(±9.47)	1.72^a^ (±0.29)	13.34^ab^(±7.75)	1.02^bc^(±0.05)	0^b^ (±0.00)	0^b^ (±0.00)
2019	Bałcyny	Floradur	43.00^abc^(±14.31)	1.34^a^ (±0.18)	1.45^b^ (±2.90)	0.25^bcd^(±0.50)	0^b^ (±0.00)	0^b^ (±0.00)
		Duranegra	47.00^abc^(±20.49)	1.44^a^ (±0.09)	0^b^ (±0.00)	0^d^ (±0.00)	0^b^ (±0.00)	0^b^ (±0.00)
		Durasol	36.25^bc^(±15.00)	1.18^a^ (±0.12)	1.00^b^ (±2.00)	0.50^bcd^(±0.10)	0^b^ (±0.00)	0^b^ (±0.00)
		Duragold	58.00^abc^(±19.18)	1.52^a^ (±0.11)	0^b^ (±0.00)	0^d^ (±0.00)	0^b^ (±0.00)	0^b^ (±0.00)
		Tamadur	44.00^abc^(±14.45)	1.23^a^ (±0.09)	0^b^ (±0.00)	0^d^ (±0.00)	0^b^ (±0.00)	0^b^ (±0.00)
		Duramonte	47.00^abc^(±17.09)	1.42^a^ (±0.17)	1.00^b^ (±2.00)	0.25^bcd^(±0.50)	0^b^ (±0.00)	0^b^ (±0.00)
		Duralis	46.00^abc^(±10.58)	1.3^a^ (±0.15)	1.00^b^ (±2.00)	0.25^bcd^(±0.50)	0^b^ (±0.00)	0^b^ (±0.00)
		Duramant	66.75^abc^(±17.23)	1.41^a^ (±0.15)	1.00^b^ (±2.00)	0.25^bcd^(±0.50)	0^b^ (±0.00)	0^b^ (±0.00)
	Niedrzwica Kościelna	Floradur	69.04^abc^(±12.67)	1.21^a^ (±0.11)	9.52^ab^(±3.12)	1.00^bc^(±0.11)	0^b^ (±0.00)	0^b^ (±0.00)
		Duranegra	43.30^abc^(±9.45)	1.15^a^ (±0.13)	10.00^ab^(±0.19)	1.00^bc^(±0.11)	0^b^ (±0.00)	0^b^ (±0.00)
		Duragold	31.25^c^ (±5.78)	1.6^a^ (±0.10)	14.60^ab^(±2.23)	1.00^bc^(±0.11)	0^b^ (±0.00)	0^b^ (±0.00)
		Tamadur	60.00^abc^(±10.67)	1.28^a^ (±0.15)	6.67^ab^(±2.51)	1.00^bc^(±0.11)	0^b^ (±0.00)	0^b^ (±0.00)
		Duralis	65.31^abc^(±5.89)	1.5^a^ (±0.09)	4.01^b^ (±1.19)	2.00^a^ (±0.51)	0^b^ (±0.00)	0^b^ (±0.00)
		Duramant	38.23^bc^(±4.19))	1.61^a^ (±0.17)	2.91^ab^(±1.41)	1.00^bc^(±0.11)	0^b^ (±0.00)	0^b^ (±0.00)
	Average for year	2018	70.19^A^(±11.37)	1.68^A^(± 0.18)	12.69^A^(±6.73)	1.10^A^(±0.21)	1.42^A^(±2.70)	0.35^A^(±0.48)
		2019	48.92^B^(±16.59)	1.36^B^(±0.16)	1.83^B^(±3.46)	0.34^B^(±0.58)	0^B^(±0.00)	0^B^(±0.00)
	Average for location	Bałcyny	62.01^Z^(±18.28)	1.56^Z^(±0.24)	8.21^Z^(±8.58)	0.69^W^(±0.58)	1.30^Z^(±2.62)	0.32^Z^(±0.47)
		Niedrzwica Kościelna	63.35^Z^(±13.60)	1.58^Z^(±0.23)	9.87^Z^ (±5.56)	1.12^Z^(±0.22)	0^W^(±0.00)	0^W^(±0.00)
	Average for cultivar	Floradur	65.57^X^(±16.66)	1.57^X^(±0.24)	8.54^Y^(±4.77)	0.90^Y^(±0.45)	2.80^Y^(±4.29)	0.48^Y^(±0.52)
		Duranegra	64.51^X^(±19.63)	1.64^X^(±0.22)	7.31^Y^(±6.19)	0.80^Y^(±0.53)	0.67^XY^(±1.54)	0.22^XY^(±0.43)
		Durasol	58.82^X^(±16.86)	1.51^XY^(±0.25)	13.,22^Y^(±9.67)	1.00^Y^(±0.54)	0.59^XY^(±1.40)	0.18^XY^(±0.39)
		Duragold	67.33^X^(±15.47)	1.62^X^(±0.17)	7.55^Y^(±5.80)	0.89^Y^(±0.50)	0.42^XY^(±0.84)	0.21^XY^(±0.42)
		Tamadur	62.87^X^(±14.76)	1.61^X^(±0.30)	12.82^Y^(±9.40)	0.84^Y^(±0.45)	0.84^XY^(±1.92)	0.21^XY^(±0.42)
		Duramonte	47.00^X^(±17.09)	1.42^XY^(±0.17)	1.00^X^(±2.00)	0.25^X^(±0.50)	0^X^(±0.00)	0^X^(±0.00)
		Duralis	49.86^X^(±12.60)	1.34^Y^(±0.16)	1.61^X^(±2.19)	0.60^XY^(±0.89)	0^X^(±0.00)	ab(±0.21)
		Duramant	61.05^X^(±19.63)	1.45^XY^(±0.16)	1.38^X^(±1.93)	0.40^X^(±0.55)	0^X^(±0.00)	0^X^(±0.00)

**Notes.**

Values followed by identical letters in columns did not differ significantly in the SNK test (*p* < 0.001).

### Koch’s postulate test

**Figure 3:**
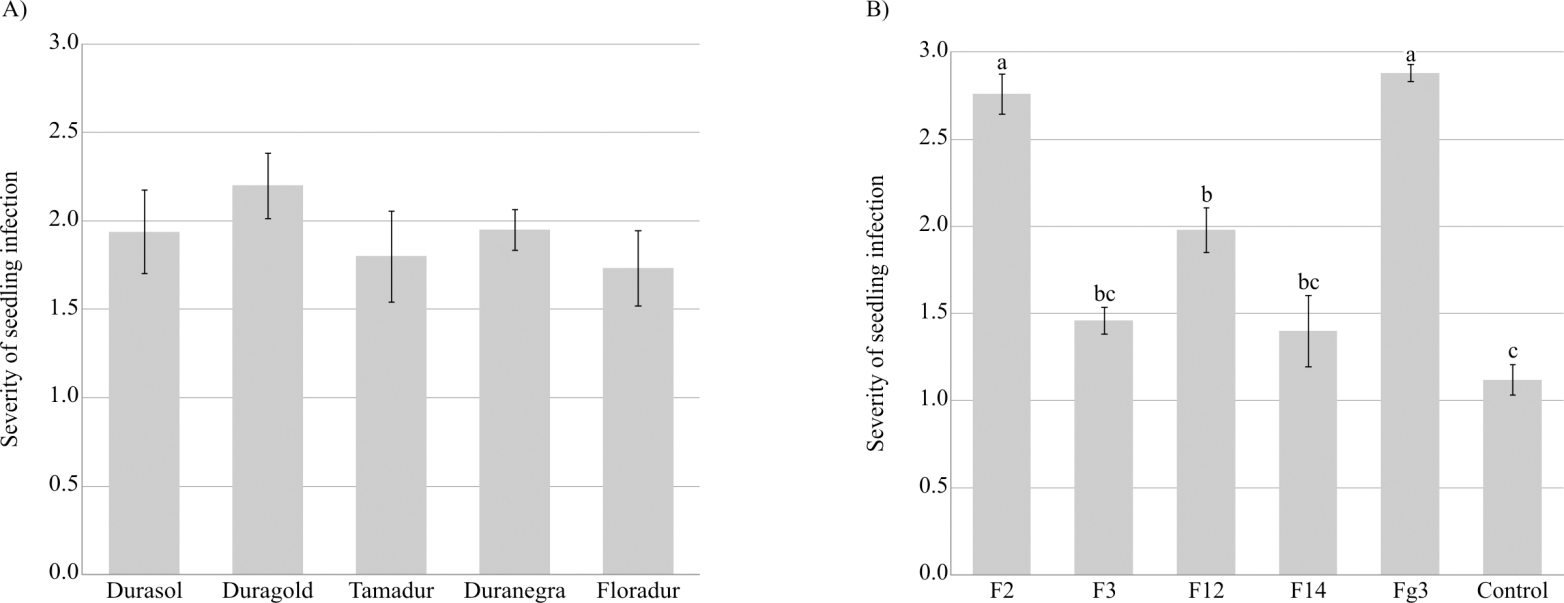
Severity of *F. graminearum.* infection depending on the tested cultivar (A) and strain (B). Values followed by identical letters did not differ significantly in the SNK test (*p* < 0.001).

*Fusarium graminearum* strains infected the roots and coleoptiles of durum wheat seedlings in Koch’s postulate test. The sequences of five isolates were compared with *F. graminearum* sequences. The compared isolates were obtained from a gene bank of plant pathogens. An analysis of the amplified ITS-I−5.8SrDNA-ITS-II region confirmed that all five isolates belonged to *F. graminearum* (sequence identity exceeded 97% in the BLASTn analysis). All seedlings were severely infected, and the tested cultivars did not differ significantly in their sensitivity to inoculation with *F. graminearum* (Fig. 3, Table S3). *Fusarium graminearum* strains Fg3 and F2 were particularly virulent for durum wheat seedlings, and in the vast majority of cases, kernels grown on PDA discs overgrown by these fungal isolates did not germinate.

### Standard seed germination test

The grain of five spring durum wheat cultivars germinated weakly, and symptoms of *Fusarium* infection were observed in 21.25–58.75% of the seedlings (Table 5, Table S3). Seed dressing with a suspension of the *D. hansenii* isolate or triticonazole decreased the percentage of ungerminated kernels, but significant differences were observed only in fungicide-treated seeds. The average efficacy of fungicide treatment in reducing the number of ungerminated kernels was estimated at 42.6% relative to untreated grain. In wheat cv. Floradur, the seed treatment involving a suspension of *D. hansenii* yeast protected 31.1% of the seedlings on average against grain pathogens. The fungicide treatment exerted phytotoxic effects on the seedings of all durum wheat cultivars. On average, fungicide-treated seeds produced shorter seedlings with significantly lower values of the normalized difference vegetation index (NDVI) relative to the control treatment. The roots of fungicide-treated seedlings were significantly longer than the roots of unprotected seedlings. In turn, biological seed dressing significantly increased the NDVI values of seedlings relative to the control treatment.

**Table 5:** Prevalence and severity of infection in selected spring cultivars of durum wheat in the blotter test.

Treatment	Cultivar	Percentage of healthy seedlings	Percentage of infected seedlings	Percentage of ungerminated kernels	Infection severity on seedlings^*^	Seedling height (cm)	Root length (cm)	NDVI^**^
Control	Tamadur	31.25^ab^(±11.81)	35.00^ab^(±7.07)	43.75^abc^(±8.54)	2.23a(±0.88)	19.28^ab^(±3.17)	19.23^ab^(±1.39)	0.486^c-g^(±0.07)
	Floradur	27.50^ab^(±17.56)	21.25^b^ (±9.46)	56.25^a^ (±16.52)	1.98a(±0.87)	27.88^a^ (±14.97)	21.00^ab^(±0.0)	0.506^b-g^(±0.06)
	Duragold	23.75^ab^(±12.50)	22.50^b^ (±8.66)	53.75^a^ (±10.31)	1.80a(±0.64)	17.85^ab^(±3.80)	18.33^b^ (±3.14)	0.554^a-d^(±0.06)
	Duranegra	12.50^b^ (±5.50)	38.75^ab^(±10.31)	41.25^abc^(±18.87)	2.40a(±0.71)	18.33^ab^(±2.94)	20.50^ab^(±1.21)	0.564^abc^(±0.04)
	Durasol	31.25^ab^(±11.09)	32.5-^ab^(±2.89)	40.00^abc^(±8.16)	2.13a(±0.60)	18.23^ab^(±1.72)	19.90^ab^(±0.50)	0.517^a−e^(±0.07)
Biol	Tamadur	15.00^b^ (±14.72)	25.00^ab^(±9.13)	50.00^ab^(±9.13)	0,85a(±0.21)	16.70^ab^(±0.96)	21.03^ab^(±5.84)	0.524^a−e^(±0.05)
	Floradur	40.00^ab^(±7.07)	16,25^b^ (±2.50)	38.75^abc^(±13.15)	1.85a(±0.19)	20.50^ab^(±2.04)	22.63^ab^(±1.65)	0.564^abc^(±0.04)
	Duragold	26.25^ab^(±19.31)	22.50^b^ (±8.66)	51.25^ab^(±11.09)	2.25a(±0.65)	17.08^ab^(±1.40)	21.50^ab^(±1.73)	0.579^ab^(±0.04)
	Duranegra	18.75^b^ (±14.93)	33.75^ab^(±7.50)	42.50^abc^(±9.57)	2.13a(±0.38)	17.23^ab^(±2.42)	21.87^ab^(±0.90)	0.591^a^ (±0.04)
	Durasol	16.25^b^ (±4.79)	28.75^ab^(±4.79)	51.25ab(±4.79)	1.90a(±0.92)	18.53^ab^(±1.44)	19.85^ab^(±1.20)	0.543^a-d^(±0.05)
Fung	Tamadur	32.50^ab^(±21.02)	36.25^ab^(±26.58)	31.25^abc^(±9.46)	1.55a(±1.39)	13.75^b^ (±3.35)	23.03^ab^(±2.08)	0.435^g^ (±0.13)
	Floradur	31.25^ab^(±20.16)	27.50^ab^(±30.69)	41.25^abc^(±27.20)	1.04a(±0.63)	11.25^b^ (±3.57)	21.24^ab^(±3.50)	0.452^fg^(±0.06)
	Duragold	56.25^a^ (±13.77)	32.50^ab^(±11.90)	18.75^bc^(±15.48)	1.53a(±0.36)	13.45^b^ (±0.64)	22.75^ab^(±1.89)	0.467^efg^(±0.07)
	Duranegra	27.50^ab^(±15.55)	58.75^a^ (±13.77)	15.00^c^ (±9.13)	2.35a(±0.47)	13.38^b^ (±0.59)	24.05^a^ (±0.25)	0.482^c-g^(±0.06)
	Durasol	27.50^ab^(±10.41)	43.75^ab^(±20.56)	28.75^abc^(±17.02)	2.05a(±0.58)	12.90^b^ (±1.52)	23.45^ab^(±0.34)	0.457^fg^(±0.10)
Average per treatment	Control	25.25^AB^(±13.03)	25.25^B^(±8.66)	47.00^A^(±13.91)	2.11^X^(±0.70)	20.31^A^(±7.51)	19.79^B^(±1.75)	0.526^B^(±0.07)
	Biol	23.25^B^(±15.15)	30.00^AB^(±10.13)	46.75^A^(±10.29)	1.89^X^(±0.67)	18.12^A^(±2.06)	21.37^AB^(±2.52)	0.560^A^(±0.05)
	Fung	35.00^A^(±18.50)	39.75^A^(±22.45)	27.00^B^(±17.87)	1.70^A^(±0.83)	12.95^B^(±2.26)	22.90^A^(±2.03)	0.459^C^(±0.09)
Average per cultivar	Tamadur	26.25^X^(±16.94)	21.67^Y^(±17.49)	41.67^X^(±11.55)	1.68^X^(±1.10)	16.56^X^(±3.56)	21.10^X^(±3.41)	0.482^Z^(±0.09)
	Floradur	32.92^X^(±15.44)	25.83^XY^(±10.19)	45.42^X^(±19.71)	1.62^X^(±0.72)	19.88^X^(±10.78)	21.62^X^(±2.15)	0.508^YZ^(±0.07)
	Duragold	35.42^X^(±20.83)	32.08^XY^(±16.02)	41.25^X^(±20.13)	1.86^X^(±0.60)	16.13^X^(±2.93)	20.86^X^(±2.88)	0.533^XY^(±0.07)
	Duranegra	19.58^X^(±13.22)	35.00^XY^(±12.97)	32.92^X^(±17.90)	2.31^X^(±0.51)	16.23^X^(±3.03)	22.16^X^(±1.78)	0.546^X^(±0.07)
	Durasol	25.00^X^(±10.66)	43.75^X^(±14.94)	40.00^X^(±13.98)	2.03^X^(±0.66)	16.55^X^(±3.01)	21.07^X^(±1.89)	0.506^YZ^(±0.08)

**Notes.**

* Average infection severity on a four-point scale.

** NDVI, Normalized Difference Vegetation Index.

Values followed by identical letters in columns did not differ significantly in the SNK test (*p* < 0.–1). Standard deviation is indicated in parentheses.

### Multivariate analysis

**Figure 4:**
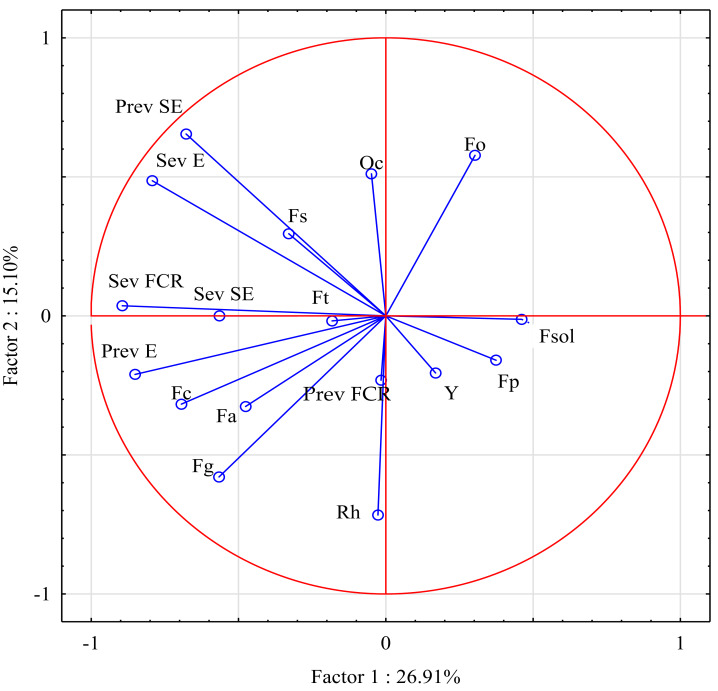
Prevalence and severity of three stem-base diseases, and the prevalence of pathogens isolated from the stem base. Fa, *Fusarium avenaceum*; Fc, *Fusarium culmorum*; Fg, *Fusarium graminearum*; Fo, *Fusarium oxysporum*; Fp, *Fusarium poae*; Fsol, *Fusarium solani*; Fs, *Fusarium sporotrichioides*; Ft, *Fusarium tricinctum*; Y, Yeast; Oc, *Oculimacula* spp; Rh, *Rhizoctonia* spp.; Prev FCR, Prevalence of Fusarium crown rot; Sev FCR, Severity of Fusarium crown rot; Prev E, Prevalence of Eyespot; Sev E, Severity of Eyespot; Prev SE, Prevalence of Sharp eyespot; Sev SE, Severity of Sharp eyespot.

In the principal component analysis, 17 variables (prevalence and severity of three stem-base diseases, and the prevalence of pathogens isolated from the stem base) explained 42.01% of the variance in both principal components (Fig. 4). The first principal component (PC1), which comprised mainly the abundance of *F. graminearum* and *Rhizoctonia* sp., explained 26.91% of the variance. The second principal component (PC2), which comprised mostly FCR severity and the prevalence of *F. culmorum* isolates, explained 15.10% of the variance. Interestingly, the severity of sharp eyespot and the prevalence of eyespot were clustered around FCR severity.

Several significant positive correlations were observed between the severity of take-all diseases and the prevalence of the isolated pathogens (Table 6). The severity of FCR was significantly correlated with the prevalence and severity of eyespot, and the prevalence of sharp eyespot (*r* = 0.776, *r* = 0.631, and *r* = 0.645, respectively). The prevalence of eyespot was significantly correlated with the severity of this disease (*r* = 0.573), and the severity of eyespot was significantly correlated with the prevalence of sharp eyespot (*r* = 0.829). In turn, FCR severity was significantly positively correlated with the prevalence of *F. culmorum* and *F. graminearum* isolates (*r* = 0.485 and *r* = 0.480, respectively). Surprisingly, the prevalence of *F. culmorum* isolates was also bound by significant positive correlations with the prevalence and severity of eyespot (*r* = 0.641 and *r* = 0.535, respectively).

## Discussion

This study was undertaken to assess the threat posed by polyphagous pathogens which infect the roots and the stem -bases of durum wheat grown in the temperate climate. In durum wheat cultivars grown in field and plot experiments, FCR was the most prevalent disease, whereas symptoms of eyespot were noted less frequently, and sharp eyespot was very rarely observed. In a study by Gorczyca et al. (2018), FCR was also the predominant disease of durum wheat grown in southern Poland. In the present experiment, symptoms of sharp eyespot were noted sporadically and in only one location (Bałcyny, in 2018), and similar results were reported by Lemańczyk & Kwaśna (2013).

In this study, durum wheat stems were colonized mainly by *F. graminearum,* which is consistent with the results reported in other Polish regions Kuzdraliński et al. (2014) and in different cereal growing areas in France (South East) (Ioos, Belhadj & Menez, 2004). *Fusarium culmorum* was isolated less frequently. In the 1990s, *F. culmorum* was the predominant stem-base pathogen of cereals in Poland, whereas *F. graminearum* was far less prevalent (Wachowska, 2000). Before 2006, only several *F. graminearum* strains had been isolated from cereals, including from rye seedlings (Kiecana, Cegiełko & Mielniczuk, 2009) and from the base of rye stems (Mielniczuk, Kiecana & Cegielko, 2012). In Poland, *F. graminearum* emerged as the dominant stem-base pathogen around 15 years ago, initially in southern Poland (Stępień et al., 2008) and, subsequently, in other Polish regions (Wachowska et al., 2023). These findings were attributed mainly to climatic conditions (Kuzdraliński et al., 2014).

**Table 6:** Correlations between the prevalence and severity of stem-base diseases and the prevalence of the isolated pathogens.

Parameter	Fa^*^	Fc	Fg	Fo	Fp	Fsol	Fs	Ft	Y	Oc	Rh	Prev FCR	Sev FCR	Prev E	Sev E	Prev SE	Sev SE
Fa	1.000																
Fc	0.272	1.000															
Fg	0.456	0.493^**^	1.000														
Fo	−0.239	−0.334	−0.340	1.000													
Fp	−0.116	−0.176	−0.315	−0.187	1.000												
Fsol	−0.147	−0.305	−0.276	0.064	−0.136	1.000											
Fs	0.098	0.106	−0.099	−0.162	−0.139	0.066	1.000										
Ft	−0.086	−0.062	0.221	−0.020	−0.080	0.020	0.007	1.000									
Y	0.286	−0.057	−0.036	−0.174	−0.130	0.519^**^	0.306	−0.125	1.000								
Oc	−0.200	−0.037	−0.095	0.145	−0.134	0.087	0.064	−0.137	−0.163	1.000							
Rh	0.051	0.321	0.519^**^	−0.196	−0.095	−0.120	−0.159	−0.070	−0.021	−0.163	1.000						
Prev FCR	0.013	0.203	0.066	−0.019	0.297	0.053	−0.170	0.339	0.019	0.087	0.207	1.000					
Sev FCR	0.465	0.485^*^	0.480^*^	−0.297	−0.319	−0.329	0.185	0.204	−0.250	0.184	−0.002	0.027	1.000				
Prev E	0.424	0.641^**^	0.450	−0.415	−0.265	−0.236	0.153	0.251	−0.095	−0.243	0.064	0.090	0.776^**^	1.000			
Sev E	0.149	0.535	0.153	0.060	−0.285	−0.390	0.453	0.198	−0.174	0.327	−0.311	0.070	0.631^**^	0.573	1.000		
Prev SE	0.195	0.225	0.006	0.278	−0.372	−0.401	0.345	0.109	−0.194	0.300	−0.412	−0.074	0.645^**^	0.444	0.829^**^	1.000	
Sev SE	0.149	0.294	0.274	−0.309	−0.123	−0.190	0.369	−0.111	−0.067	−0.104	−0.098	−0.205	0.461	0.453	0.352	0.268	1.000

**Notes.**

* Fa, *Fusarium avenaceum*; Fc, *Fusarium culmorum*; Fg, *Fusarium graminearum*; Fo, *Fusarium oxysporum*; Fp, *Fusarium poae*; Fsol, *Fusarium solani*; Fs, *Fusarium sporotrichioides*; Ft, *Fusarium tricinctum*; Y, Yeast; Oc, *Oculimacula* spp.; Rh, *Rhizoctonia* spp.; Prev FCR, Prevalence of Fusarium crown rot; Sev FCR, Severity of Fusarium crown rot; Prev E, Prevalence of Eyespot; Sev E, Severity of Eyespot; Prev SE, Prevalence of Sharp eyespot; Sev SE, Severity of Sharp eyespot.

** Significant difference at *p* < 0.001; **-, significant difference at *p* < 0.005.

In the current study, a significant positive correlation was noted between FCR severity in experimental plots and the prevalence of *F. graminearum* and *F. culmorum* strains isolated from infected stems of durum wheat. In the work of Saad et al. (2022), *F. culmorum* caused significantly greater discoloration of durum wheat stems than *F. pseudograminearum* in a pathogenicity test (stems were inoculated with the tested pathogens in experimental plots). The results of Koch’s test conducted by Wang & Gottwald (2017) (roots were inoculated in a climate chamber) suggested that root colonization by *F. graminearum* can lead to systemic plant invasion under field conditions. The present study demonstrated that some *F. graminearum* strains could be highly virulent for durum wheat seedlings and could induce necrosis in the earliest stages of seedling development. Wang & Gottwald (2017) also reported that the colonization of common wheat roots by *F. graminearum* negatively affected plant development and led to systemic plant invasion. *Fusarium graminearum* is a hemibiotrophic pathogen with a unique infection strategy that involves complex, specialized structures and processes (Wang et al., 2018).

*Fusarium avenaceum* was also frequently isolated from winter durum wheat cvs. Wintergold and Karmadur in selected locations (fields No. 12 and 14). According to Šišić et al. (2018), this pathogen readily colonizes soils with a low content of organic matter and nutrients. *Fusarium avenaceum* has emerged as the dominant pathogen of cereals in Norway (Yli-Mattila, 2010). In the present study, *F. oxysporum* was isolated from durum wheat in eight agricultural fields and in experimental plots in southern Poland. In a field trial conducted in Nebraska, *F. oxysporum* and *F. graminearum* were the most prevalent causative agents of root rot in maize, soybeans, and wheat (Parikh et al., 2018). There is considerable evidence to indicate that the prevalence of *Fusarium* pathogens is closely correlated with edaphic factors, climate, and the species of preceding crops (Moparthi et al., 2021). In a study by Gorczyca et al. (2018), the prevalence of FCR was highest in a dry growing season. According to Korbas & Mrówczyński (2009), unlike other stem-base diseases, the progression of FCR does not require moist conditions. In addition, one *F. culmorum* strain and one *F. graminearum* strain isolated from chickpeas and lentil seeds by Moparthi et al. (2021) were highly virulent for wheat. These observations suggest that even non-cereal preceding crops can be reservoirs of *Fusarium* strains that are virulent for durum wheat. In turn, crop rotation significantly decreased the prevalence of two pathogens of durum wheat grown in Canada: *Bipolaris sorokiniana* (teleomorph: *Cochliobolus sativus*) was less abundant in durum wheat following one year of peas, whereas *Fusarium torulosum* was less prevalent following canola crops (Vujanovic, Mavragani & Hamel, 2012). Nitrogen (N) fertilization can also modify the prevalence of FCR. In a study by Buster et al. (2023), the *in vitro* growth of *F. pseudograminearum* colonies was accelerated in the presence of N, and a greater increase was reported when N was supplied as urea than as ammonium nitrate. In the cited study, the inoculation of durum wheat with the pathogen causing FCR decreased N transport in stems by 10.2% relative to non-inoculated plants.

In the current study, durum wheat grain harvested in 2020 was abundantly colonized by *Fusarium* spp. (Fig. S4), and these kernels germinated weakly in the blotter test. Seed dressing with a triazole fungicide was not highly effective in reducing the number of ungerminated kernels (average efficiency—42.6%), and in cv. Floradur, seed treatment with a suspension of *Debaryomyces hansenii* yeast protected 31.1% of the seedlings on average against grain pathogens. Similar results were reported by Moya-Elizondo & Jacobsen (2016) in a greenhouse experiment, where seed dressing with difenoconazole and mefenoxam fungicides decreased the severity of FCR in common wheat seedings by 29–50%. In the cited study, seed treatment with *Bacillus pumilis* 314-16-5 and *Trichoderma harzianum* T-22 isolates was significantly less effective in reducing FCR severity. Moya-Elizondo & Jacobsen (2016) also reported an increase in the activity of three pathogenesis-related (PR) proteins: peroxidase, endochitinase, and *β*-1,3-glucanase, in biologically protected plants. The increase in enzyme activity differed subject to wheat cultivar and antagonist species. In the work of Kthiri et al. (2020), the prevalence of FCR in durum wheat plants protected with *Trichoderma* strains or Trianum-T22, a *Trichoderma*-based commercial preparation, decreased by 35.59% and 51.79%, respectively, relative to the control treatment, 20 days after inoculation with *F. culmorum*. The cited authors also observed an increase in the content of phenolic compounds and peroxidase activity in biologically protected plants. These findings indicate that biological preparations significantly affect plant defense mechanisms and that their effectiveness varies across wheat cultivars. Triazole fungicides have a different mechanism of action that directly targets pathogens (Liu et al., 2023). Triticonazole, a 14*α*-demethylation inhibitor (DMI) targeting fungal ergosterol biosynthesis, has been registered as a seed treatment for protecting wheat against stem-base diseases. In the present study, triticonazole was more effective in reducing the symptoms of FCR than the biological treatment, but it also exerted inhibitory/phytotoxic effects on seedlings. Zhang et al. (2020) examined maize seedlings grown from seeds dressed with triazole fungicides and concluded that these effects could be attributed to an imbalance of gibberellin GA12 and abscisic acid and the up-regulation of two catabolic enzyme genes under chilling stress.

In the present study, durum wheat cultivars grown in field and plot experiments, and the cultivars examined in Koch’s test were severely infected with *Fusarium* fungi, in particular *F. graminearum*, and German and Slovakian cultivars did not differ significantly in their susceptibility to infection. Bouanaka, Bellil & Khelifi (2021) also reported the absence of significant differences in susceptibility to inoculation with *F. culmorum* between eight durum wheat cultivars grown in Algeria. In the cited study, the results of Koch’s postulate test (with a similar design to that used in the present study) demonstrated that the pathogen produced large mycelia that came into direct contact with germinating kernels and were not affected by environmental constraints. In a more recent study, Bouarda et al. (2022) analyzed the susceptibility of 20 durum wheat genotypes to *F. graminearum* by applying fungal spores to seedling roots. At lower levels of pathogen pressure, the studied genotypes were classified as resistant (for example, cv. Marouane was resistant to three *F. culmorum* strains), moderately resistant, moderately susceptible, and susceptible. However, none of the tested wheat genotypes was resistant to all pathogenic strains (Bouarda et al., 2022).

Deoxynivalenol (DON) is one of the virulence factors of *F. culmorum* and *F. graminearum.* Deoxynivalenol does not play a key role in the initial stage of stem infection, but *Fusarium* strains with a mutation of the gene encoding DON were less virulent (Mudge et al., 2006; Scherm et al., 2011). According to Mandalà et al. (2019), DON plays a key role in the progression of fungal infections in cereal stems. In wheat, uridine diphosphate-dependent glucosyltransferases (UGTs) transform DON to DON-3-*β*-d-glucoside (D3G) (Berthiller et al., 2005; Bowles et al., 2006). Transgenic Ubi-UGT durum wheat lines were less susceptible to *F. culmorum* infection (Mandalà et al., 2019), but pyramiding pectin methylesterase inhibitors (PMEIs) did not enhance seedling resistance to FCR caused by *F. graminearum* (Mandalà et al., 2021).

## Conclusions

The production of durum wheat poses a challenge in the temperate climate because this wheat species is susceptible to infections caused by *Fusarium* fungi, in particular *F. graminearum*. This is the first study to investigate the prevalence and severity of stem-base diseases in Slovakian and German cultivars of durum wheat grown in different Polish regions, and to analyze the virulence of *F. graminearum* for durum wheat seedlings. Integrated biological and chemical seed treatments and the selection of the most resistant durum wheat cultivar did not produce the anticipated results: seed treatments did not reduce infection severity, and none of the tested cultivars was less susceptible to infection. In addition, the prevalence of FCR was strongly correlated with the year of study and location, which suggests that stem-base health is influenced by uncontrolled and unidentified factors. In the future, additional durum wheat cultivars and seed treatments will be included in the analysis.

##  Supplemental Information

10.7717/peerj.20105/supp-1 Supplemental Information 1 One-way ANOVA of the prevalence and severity of stem-base diseases in the fields

10.7717/peerj.20105/supp-2 Supplemental Information 2 Three-way ANOVA of the prevalence and severity of stem-base diseases

10.7717/peerj.20105/supp-3 Supplemental Information 3 Two-way ANOVA of the prevalence and severity of infection in selected spring cultivars of durum wheat in the blotter test

10.7717/peerj.20105/supp-4 Supplemental Information 4 Prevalence and severity of infection in selected spring cultivars of durum wheat in the blotter test

10.7717/peerj.20105/supp-5 Supplemental Information 5 Symptoms (A, B) - Fusarium crown rot (FCR) (Fusarium spp.), (C, D) - eyespot (Oculimacula spp.), and (E) - sharp eyespot (Rhizoctonia spp.) in early (A, C, E) and late stages of development of durum wheat (A, C, E) and symptoms of infection of roots and coleoptile of durum wheat seedlings by *Fusarium graminearum.*

10.7717/peerj.20105/supp-6 Supplemental Information 6 Raw data for Fig. 3

10.7717/peerj.20105/supp-7 Supplemental Information 7 Raw data for Table 2

10.7717/peerj.20105/supp-8 Supplemental Information 8 Raw data for Table 4

10.7717/peerj.20105/supp-9 Supplemental Information 9 Raw data for Table 5

10.7717/peerj.20105/supp-10 Supplemental Information 10 Raw data for Table 5A

## Additional Information and Declarations

### Field Study Permissions

The following information was supplied relating to field study approvals (*i.e.*, approving body and any reference numbers): The trials were localized in the area of Experimental Unit in Bałcyny which belongs to the Warmia and Mazury University in Olsztyn. The trials in Niedrzwica Kościelna were conducted on the private crop fields. The payment for these trials were realized in frame of project which was managed by Mr Dr Gontarz, who is one of co-authors.

### Data Availability

The following information was supplied regarding data availability: The data is available at GenBank: F2 (MZ827460), F3 (MZ827461), F12 (MZ827462), F14 (MZ827463), and Fg3 (MZ820057). The data is available in the Supplemental Files.
