# Supplementary material for: Screening durum wheat cultivars for resistance traits against the stem-base pathogen Fusarium graminearum
Source: PeerJ. 2025 Nov 13;13:e20105. doi: 10.7717/peerj.20105 (PMC12619947; doi:10.7717/peerj.20105)
Supplement: Supplemental Information 1 [file peerj-13-20105-s001.docx]

| Table S1. One-way ANOVA of the prevalence and severity of stem-base diseases | | | | | |
| --- | --- | --- | --- | --- | --- |
| Factor | df | Fusarium crown rot | | Eyespot | |
|  |  | Prevalence | Severity | Prevalence | Severity |
|  |  | F | | | |
| Location | 23 | 116.31** | 26.34** | 393.99** | 35.21** |
| *significant difference at p < 0.001; ** - significant difference at p < 0.005. | | | | | |
