# Supplementary material for: Screening durum wheat cultivars for resistance traits against the stem-base pathogen Fusarium graminearum
Source: PeerJ. 2025 Nov 13;13:e20105. doi: 10.7717/peerj.20105 (PMC12619947; doi:10.7717/peerj.20105)
Supplement: Supplemental Information 2 [file peerj-13-20105-s002.doc]

| Table S2. Three-way ANOVA of the prevalence and severity of stem-base diseases | | | | | | |  |
| --- | --- | --- | --- | --- | --- | --- | --- |
| Factor | df | Fusarium crown rot | | Eyespot | | Sharp eyespot | |
|  |  | Prevalence | Severity | Prevalence | Severity | Prevalence | Severity |
|  |  | F | | | | | |
| Year (Y) | 1 | 33.09** | 45.02** | 33.58** | 26.59** | 5.42** | 12.92** |
| Location (L) | 1 | 0.34 | 0.48 | 1.48 | 35.21** | 5.41** | 12.91** |
| Cultivar (Cv) | 3 | 0.57 | 1.15 | 0.64 | 0.22 | 1 | 0.42 |
| YxL | 1 | 1.87 | 2.36 | 16.59** | 35.01* | 5.42** | 12.91** |
| YxCv | 3 | 0.56 | 3.14* | 2.89* | 0.06 | 1 | 0.42 |
| LxCv | 3 | 3.37* | 1.34 | 0.46 | 0.19 | 1 | 0.42 |
| YxLxCv | 3 | 2.55 | 0.4 | 0.9 | 0.4 | 1 | 0.42 |
| *significant difference at p < 0.001; ** - significant difference at p < 0.005. | | | | | | | |
