# Supplementary material for: Screening durum wheat cultivars for resistance traits against the stem-base pathogen Fusarium graminearum
Source: PeerJ. 2025 Nov 13;13:e20105. doi: 10.7717/peerj.20105 (PMC12619947; doi:10.7717/peerj.20105)
Supplement: Supplemental Information 3 [file peerj-13-20105-s003.doc]

Table S3. Two-way ANOVA of the prevalence and severity of infection in selected spring cultivars of durum wheat in the blotter test

| Factor | df | Percentage of healthy seedlings | Percentage of infected seedlings | Percentage of ungerminated kernels | Infection severity on seedlings* | Seedling height (cm) | Root length (cm) | NDVI** |
| --- | --- | --- | --- | --- | --- | --- | --- | --- |
| Treatment (T) | 2 | 3.92* | 5.51* | 14.06** | 1.69 | 13.55** | 10.79** | 47.73** |
| Cultivar (Cv) | 4 | 2.41 | 4.41* | 1.34 | 1.91 | 1.40 | 0.68 | 6.85** |
| TxCv | 8 | 1.90 | 0.33 | 1.45 | 1.06 | 1.35 | 1.10 | 0.39 |
