# Supplementary material for: Screening durum wheat cultivars for resistance traits against the stem-base pathogen Fusarium graminearum
Source: PeerJ. 2025 Nov 13;13:e20105. doi: 10.7717/peerj.20105 (PMC12619947; doi:10.7717/peerj.20105)
Supplement: Supplemental Information 5 — (A, B) - Fusarium crown rot (FCR) (Fusarium spp.), (C, D) - eyespot (Oculimacula spp.), and (E) - sharp eyespot (Rhizoctonia spp.) in early (A, C, E) and late stages of development of durum wheat (A, C, E) and symptoms of infection of roots and coleoptile of durum wheat seedlings by Fusarium graminearum. [file peerj-13-20105-s005.pdf]

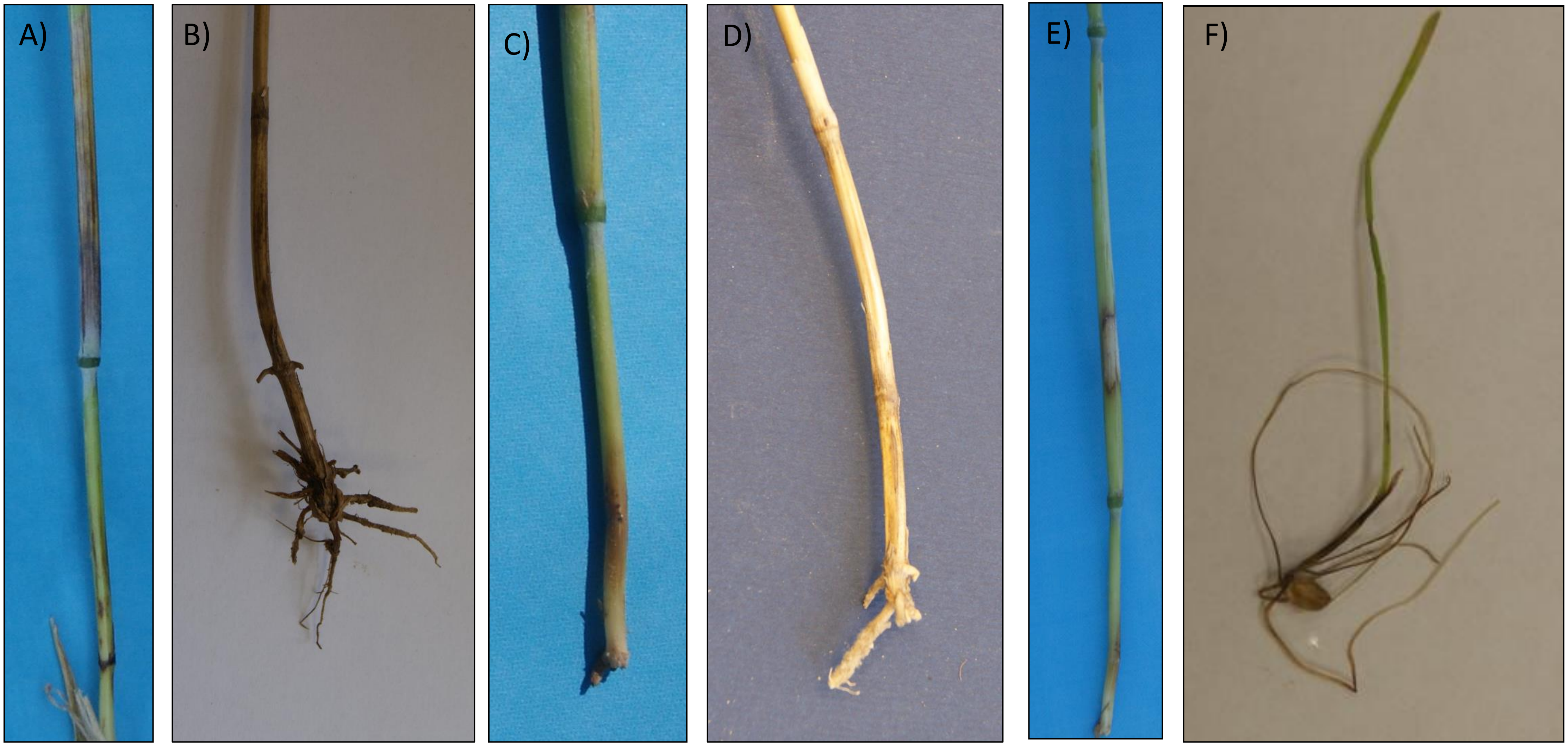

Figure S1. Symptoms A, B - Fusarium crown rot (FCR) (*Fusarium* spp.), C,D - eyespot (*Oculimacula* spp.), and E - sharp eyespot (*Rhizoctonia* spp.) in early (A,C,E) and late stages of development of durum wheat (A,C,E) and symptoms of infection of roots and coleoptile of durum wheat seedlings by *Fusarium graminearum*.
