# Supplementary material for: Screening durum wheat cultivars for resistance traits against the stem-base pathogen Fusarium graminearum
Source: PeerJ. 2025 Nov 13;13:e20105. doi: 10.7717/peerj.20105 (PMC12619947; doi:10.7717/peerj.20105)
Supplement: Supplementary file 6 [file peerj-13-20105-s006.odt]

| Cultivar | Strains | Sum | Severity FCR |
| --- | --- | --- | --- |
| Durasol | Fg4 | 18 | 3,000 |
| Durasol | Fg4 | 18 | 3,000 |
| Duragold | Fg4 | 18 | 3,000 |
| Duragold | Fg4 | 18 | 3,000 |
| Tamadur | Fg4 | 14 | 2,333 |
| Tamadur | Fg4 | 18 | 3,000 |
| Duranegra | Fg4 | 18 | 3,000 |
| Duranegra | Fg4 | 7 | 1,167 |
| Floradur | Fg4 | 18 | 3,000 |
| Floradur | Fg4 | 18 | 3,000 |
| Durasol | Fg 39 | 8 | 1,333 |
| Durasol | Fg 39 | 12 | 2,000 |
| Duragold | Fg 39 | 9 | 1,500 |
| Duragold | Fg 39 | 9 | 1,500 |
| Tamadur | Fg 39 | 8 | 1,333 |
| Tamadur | Fg 39 | 8 | 1,333 |
| Duranegra | Fg 39 | 6 | 1,000 |
| Duranegra | Fg 39 | 10 | 1,667 |
| Floradur | Fg 39 | 10 | 1,667 |
| Floradur | Fg 39 | 6 | 1,000 |
| Durasol | Fg 57 | 15 | 2,500 |
| Durasol | Fg 57 | 12 | 2,000 |
| Duragold | Fg 57 | 14 | 2,333 |
| Duragold | Fg 57 | 10 | 1,667 |
| Tamadur | Fg 57 | 12 | 2,000 |
| Tamadur | Fg 57 | 12 | 2,000 |
| Duranegra | Fg 57 | 16 | 2,667 |
| Duranegra | Fg 57 | 9 | 1,500 |
| Floradur | Fg 57 | 7 | 1,167 |
| Floradur | Fg 57 | 9 | 1,500 |
| Durasol | Fg 19 | 12 | 2,000 |
| Durasol | Fg 19 | 8 | 1,333 |
| Duragold | Fg 19 | 10 | 1,667 |
| Duragold | Fg 19 | 14 | 2,333 |
| Tamadur | Fg 19 | 6 | 1,000 |
| Tamadur | Fg 19 | 4 | 0,667 |
| Duranegra | Fg 19 | 10 | 1,667 |
| Duranegra | Fg 19 | 11 | 1,833 |
| Floradur | Fg 19 | 3 | 0,500 |
| Floradur | Fg 19 | 2 | 0,333 |
| Durasol | Fg 38 | 18 | 3,000 |
| Durasol | Fg 38 | 18 | 3,000 |
| Duragold | Fg 38 | 18 | 3,000 |
| Duragold | Fg 38 | 16 | 2,667 |
| Tamadur | Fg 38 | 17 | 2,833 |
| Tamadur | Fg 38 | 17 | 2,833 |
| Duranegra | Fg 38 | 17 | 2,833 |
| Duranegra | Fg 38 | 17 | 2,833 |
| Floradur | Fg 38 | 18 | 3,000 |
| Floradur | Fg 38 | 18 | 3,000 |
| Durasol | Control | 6 | 1,000 |
| Durasol | Control | 5 | 0,833 |
| Duragold | Control | 6 | 1,000 |
| Duragold | Control | 7 | 1,167 |
| Tamadur | Control | 4 | 0,667 |
| Tamadur | Control | 5 | 0,833 |
| Duranegra | Control | 7 | 1,167 |
| Duranegra | Control | 8 | 1,333 |
| Floradur | Control | 14 | 2,333 |
| Floradur | Control | 3 | 0,500 |
