# Supplementary material for: Screening durum wheat cultivars for resistance traits against the stem-base pathogen Fusarium graminearum
Source: PeerJ. 2025 Nov 13;13:e20105. doi: 10.7717/peerj.20105 (PMC12619947; doi:10.7717/peerj.20105)
Supplement: Supplementary file 7 [file peerj-13-20105-s007.odt]

| Cultivar (Location) | FCR prevalence | FCR severity | Eyespot prevalence | Eyespot severity |
| --- | --- | --- | --- | --- |
| Auradur (2) | 52 | 1.5 | 30 | 1.4 |
| Auradur (2) | 49 | 2.1 | 29 | 1.5 |
| Auradur (2) | 51 | 1.1 | 31 | 1.3 |
| Tempodur (12) | 58 | 1.93 | 16 | 1.37 |
| Tempodur (12) | 59 | 1.8 | 15 | 1.36 |
| Tempodur (12) | 56 | 1.7 | 17 | 1.35 |
| Wintergold (12) | 20 | 1.8 | 24 | 1.17 |
| Wintergold (12) | 19 | 1.7 | 25 | 1.16 |
| Wintergold (12) | 17 | 1.6 | 23 | 1.18 |
| Tamadur (12) | 16 | 1 | 8 | 1.25 |
| Tamadur (12) | 13 | 0.9 | 7 | 1.2 |
| Tamadur (12) | 17 | 0.99 | 7 | 1.3 |
| Elsadur (13) | 38 | 1.47 | 18 | 1 |
| Elsadur (13) | 37 | 1.5 | 17 | 0.9 |
| Elsadur (13) | 36 | 1.4 | 24 | 0.89 |
| Wintergold (12) | 18 | 1 | 36 | 1.05 |
| Wintergold (12) | 17 | 0.9 | 34 | 0.9 |
| Wintergold (12) | 28 | 0.97 | 32 | 0.89 |
| Floradur (11) | 34 | 1.59 | 4 | 1 |
| Floradur (11) | 21 | 1.5 | 3 | 0.9 |
| Floradur (11) | 33 | 1.3 | 5 | 0.89 |
| Tamadur (12) | 30 | 1.2 | 10 | 1 |
| Tamadur (12) | 29 | 1.1 | 9 | 0.9 |
| Tamadur (12) | 31 | 0.9 | 11 | 0.8 |
| Duranegra (4) | 28 | 1.86 | 0 | 0 |
| Duranegra (4) | 27 | 1.8 | 0 | 0 |
| Duranegra (4) | 26 | 1.78 | 0 | 0 |
| Duranegra (5) | 16 | 1 | 14 | 1 |
| Duranegra (5) | 15 | 0.9 | 13 | 0.9 |
| Duranegra (5) | 17 | 0.89 | 15 | 0.89 |
| Pentadur (15) | 36 | 1.55 | 10 | 1.2 |
| Pentadur (15) | 34 | 1.5 | 9 | 1.2 |
| Pentadur (15) | 35 | 1.4 | 11 | 0.9 |
| Pentadur (16) | 18 | 1.2 | 8 | 1 |
| Pentadur (16) | 17 | 1 | 7 | 0.9 |
| Pentadur (16) | 12 | 1.1 | 6 | 1.1 |
| Duragold (3) | 34 | 2.35 | 8 | 1.25 |
| Duragold (3) | 33 | 2.3 | 7 | 1.2 |
| Duragold (3) | 35 | 2.1 | 9 | 1.3 |
| Spiradur (7) | 54 | 2.26 | 2 | 2 |
| Spiradur (7) | 53 | 2.2 | 2 | 2 |
| Spiradur (7) | 52 | 2 | 1 | 1.9 |
| Duranegra (6) | 42 | 1.81 | 12 | 1.17 |
| Duranegra (6) | 40 | 1.8 | 11 | 1.16 |
| Duranegra (6) | 41 | 1.7 | 12 | 1.17 |
| Duranegra (8) | 38 | 1.79 | 4 | 1 |
| Duranegra (8) | 37 | 1.7 | 4 | 1 |
| Duranegra (8) | 34 | 1.8 | 3 | 0.9 |
| Floradur (8) | 44 | 1.59 | 8 | 1.25 |
| Floradur (8) | 44 | 1.6 | 7 | 1.2 |
| Floradur (8) | 43 | 1.7 | 8 | 1.3 |
| Durasol (8) | 26 | 2.31 | 16 | 1.5 |
| Durasol (8) | 25 | 2.3 | 14 | 1.4 |
| Durasol (8) | 24 | 2.1 | 14 | 1.3 |
| Karmadur (14) | 42 | 1.76 | 12 | 1.17 |
| Karmadur (14) | 40 | 1.77 | 10 | 1.16 |
| Karmadur (14) | 41 | 1.6 | 11 | 1.5 |
| Duranegra (9) | 66 | 2.3 | 8 | 1.25 |
| Duranegra (9) | 65 | 2.1 | 7 | 1.3 |
| Duranegra (9) | 64 | 2 | 8 | 1.2 |
| Spiradur (16) | 28 | 1.71 | 26 | 1.69 |
| Spiradur (16) | 27 | 1.7 | 25 | 1.6 |
| Spiradur (16) | 26 | 1.6 | 24 | 1.5 |
| Spiradur (16) | 24 | 1.17 | 4 | 1 |
| Spiradur (16) | 23 | 1.1 | 3 | 0.9 |
| Spiradur (16) | 22 | 1 | 4 | 1.1 |
| Duranegra (10) | 30 | 1.13 | 2 | 1 |
| Duranegra (10) | 29 | 1.1 | 2 | 0.9 |
| Duranegra (10) | 31 | 1.2 | 2 | 1 |
| Tamadur (10) | 62 | 1.58 | 62 | 1.1 |
| Tamadur (10) | 60 | 1.6 | 63 | 1 |
| Tamadur (10) | 62 | 1.5 | 61 | 0.9 |
