# Supplementary material for: Screening durum wheat cultivars for resistance traits against the stem-base pathogen Fusarium graminearum
Source: PeerJ. 2025 Nov 13;13:e20105. doi: 10.7717/peerj.20105 (PMC12619947; doi:10.7717/peerj.20105)
Supplement: Supplementary file 8 [file peerj-13-20105-s008.odt]

| Location | Cultivar | FCR prevalence | FCR severity | Eyespot prevalence | Eyespot severity | Sharp eyespot prevalence | Sharp eyespot severity |
| --- | --- | --- | --- | --- | --- | --- | --- |
| Bałcyny | Floradur | 80.4 | 1.67 | 13 | 1 | 2.2 | 1 |
| Bałcyny | Floradur | 79.6 | 1.74 | 6.5 | 1 | 4.3 | 1 |
| Bałcyny | Floradur | 88.6 | 1.67 | 9.3 | 1 | 2.3 | 1 |
| Bałcyny | Floradur | 57.4 | 1.69 | 14.9 | 1 | 4.3 | 1 |
| Bałcyny | Duranegra | 94 | 1.89 | 0 | 0 | 0 | 0 |
| Bałcyny | Duranegra | 69.6 | 1.81 | 21.7 | 1.1 | 0 | 0 |
| Bałcyny | Duranegra | 78.7 | 1.68 | 8.5 | 1.25 | 2.1 | 1 |
| Bałcyny | Duranegra | 95.6 | 1.91 | 4.35 | 1 | 0 | 0 |
| Bałcyny | Durasol | 68.7 | 1.62 | 14.6 | 1 | 4.17 | 1 |
| Bałcyny | Durasol | 78 | 1.49 | 12 | 1.33 | 0 | 0 |
| Bałcyny | Durasol | 52.1 | 1.37 | 30.5 | 1.2 | 0 | 0 |
| Bałcyny | Durasol | 62 | 1.53 | 28 | 1.5 | 2 | 1 |
| Bałcyny | Duragold | 70.5 | 1.71 | 13.6 | 1.33 | 0 | 0 |
| Bałcyny | Duragold | 72 | 1.42 | 14 | 1.14 | 2 | 1 |
| Bałcyny | Duragold | 78.9 | 1.8 | 10.5 | 1.5 | 0 | 0 |
| Bałcyny | Duragold | 75.5 | 1.79 | 6.7 | 1 | 0 | 0 |
| Bałcyny | Tamadur | 78 | 1.74 | 10 | 1.2 | 2 | 1 |
| Bałcyny | Tamadur | 54.1 | 1.76 | 27 | 1 | 0 | 0 |
| Bałcyny | Tamadur | 48 | 1.88 | 22 | 1 | 6 | 1 |
| Bałcyny | Tamadur | 64 | 1.58 | 18 | 1.1 | 6 | 1 |
| Bałcyny | Floradur | 74 | 1.73 | 8 | 1.2 | 12 | 1 |
| Bałcyny | Floradur | 84 | 1.67 | 12 | 1.3 | 4 | 1 |
| Bałcyny | Floradur | 70 | 1.94 | 8.1 | 1 | 14.2 | 1.14 |
| Bałcyny | Floradur | 70.2 | 1.47 | 12.7 | 1 | 4.3 | 1 |
| Bałcyny | Duranegra | 74 | 1.84 | 14 | 1 | 2 | 1 |
| Bałcyny | Duranegra | 82 | 1.78 | 12 | 1 | 0 | 0 |
| Bałcyny | Duranegra | 86 | 1.9 | 6 | 1 | 2 | 1 |
| Bałcyny | Duranegra | 68 | 1.8 | 14 | 1.14 | 6 | 1 |
| Bałcyny | Durasol | 74 | 1.64 | 6 | 1 | 4 | 1 |
| Bałcyny | Durasol | 78 | 1.89 | 16 | 1.38 | 0 | 0 |
| Bałcyny | Durasol | 65.9 | 1.71 | 25.5 | 1.1 | 0 | 0 |
| Bałcyny | Durasol | 60 | 1.73 | 20 | 1.3 | 0 | 0 |
| Bałcyny | Duragold | 66 | 1.79 | 10 | 1.2 | 2 | 1 |
| Bałcyny | Duragold | 70 | 1.74 | 14 | 1 | 2 | 1 |
| Bałcyny | Duragold | 79.6 | 1.85 | 14.3 | 1 | 0 | 0 |
| Bałcyny | Duragold | 80 | 1.47 | 12 | 1.33 | 2 | 1 |
| Bałcyny | Tamadur | 68 | 1.58 | 18 | 1.2 | 2 | 1 |
| Bałcyny | Tamadur | 67.4 | 1.98 | 23.9 | 1 | 0 | 0 |
| Bałcyny | Tamadur | 82 | 1.66 | 14 | 1 | 0 | 0 |
| Bałcyny | Tamadur | 68 | 1.94 | 24 | 1.3 | 0 | 0 |
| NK | Duranegra | 56 | 1.53 | 8 | 1 | 0 | 0 |
| NK | Duranegra | 62.8 | 1.48 | 6.9 | 1.3 | 0 | 0 |
| NK | Duranegra | 68.2 | 1.72 | 4.5 | 1 | 0 | 0 |
| NK | Duranegra | 45 | 1.69 | 8 | 1.5 | 0 | 0 |
| NK | Duranegra | 50 | 1.54 | 13.6 | 1.17 | 0 | 0 |
| NK | Durasol | 50.9 | 1.26 | 5.7 | 1 | 0 | 0 |
| NK | Durasol | 56.6 | 1.58 | 15.1 | 1.13 | 0 | 0 |
| NK | Durasol | 76.7 | 1.83 | 20 | 1 | 0 | 0 |
| NK | Durasol | 56.6 | 1.7 | 15.1 | 1.13 | 0 | 0 |
| NK | Durasol | 75.4 | 1.57 | 12.2 | 1 | 0 | 0 |
| NK | Duragold | 58 | 1.66 | 8 | 1 | 0 | 0 |
| NK | Duragold | 54.7 | 1.28 | 13.2 | 1.14 | 0 | 0 |
| NK | Duragold | 68.1 | 1.65 | 2.1 | 1 | 0 | 0 |
| NK | Duragold | 73.9 | 1.65 | 6.5 | 1.33 | 0 | 0 |
| NK | Duragold | 92.4 | 1.79 | 1.9 | 1 | 0 | 0 |
| NK | Duragold | 76.4 | 1.41 | 1.96 | 1 | 0 | 0 |
| NK | Tamadur | 73.9 | 1.7 | 4.35 | 1 | 0 | 0 |
| NK | Tamadur | 82.6 | 1.51 | 20 | 1 | 0 | 0 |
| NK | Tamadur | 69.2 | 1.96 | 12.8 | 1 | 0 | 0 |
| NK | Tamadur | 59.4 | 2.15 | 25 | 1.13 | 0 | 0 |
| NK | Tamadur | 81.1 | 1.4 | 9.4 | 1 | 0 | 0 |
| NK | Tamadur | 62.8 | 1.57 | 8.51 | 1 | 0 | 0 |
| NK | Floradur | 73.1 | 1.32 | 9.6 | 1 | 0 | 0 |
| NK | Floradur | 74.4 | 1.5 | 11.6 | 1.17 | 0 | 0 |
| NK | Floradur | 69.2 | 1.78 | 10.3 | 1.5 | 0 | 0 |
| NK | Floradur | 52.8 | 1.95 | 13.9 | 1.2 | 0 | 0 |
| Bałcyny | Duramonte | 60 | 1.6 | 0 | 0 | 0 | 0 |
| Bałcyny | Duramonte | 60 | 1.5 | 4 | 1 | 0 | 0 |
| Bałcyny | Duragold | 72 | 1.38 | 0 | 0 | 0 | 0 |
| Bałcyny | Duragold | 36 | 1.55 | 0 | 0 | 0 | 0 |
| Bałcyny | Tamadur | 32 | 1.11 | 0 | 0 | 0 | 0 |
| Bałcyny | Tamadur | 39 | 1.33 | 0 | 0 | 0 | 0 |
| Bałcyny | Floradur | 63 | 1.25 | 0 | 0 | 0 | 0 |
| Bałcyny | Floradur | 40 | 1.3 | 0 | 0 | 0 | 0 |
| Bałcyny | Duralis | 60 | 1.47 | 0 | 0 | 0 | 0 |
| Bałcyny | Duralis | 36 | 1.11 | 0 | 0 | 0 | 0 |
| Bałcyny | Duramant | 43 | 1.2 | 0 | 0 | 0 | 0 |
| Bałcyny | Duramant | 68 | 1.53 | 0 | 0 | 0 | 0 |
| Bałcyny | Duranegra | 20 | 1.4 | 0 | 0 | 0 | 0 |
| Bałcyny | Duranegra | 44 | 1.45 | 0 | 0 | 0 | 0 |
| Bałcyny | Durasol | 57 | 1.25 | 0 | 0 | 0 | 0 |
| Bałcyny | Durasol | 23 | 1.2 | 0 | 0 | 0 | 0 |
| Bałcyny | Duramonte | 44 | 1.36 | 0 | 0 | 0 | 0 |
| Bałcyny | Duramonte | 24 | 1.2 | 0 | 0 | 0 | 0 |
| Bałcyny | Duragold | 76 | 1.65 | 0 | 0 | 0 | 0 |
| Bałcyny | Duragold | 48 | 1.5 | 0 | 0 | 0 | 0 |
| Bałcyny | Tamadur | 65 | 1.26 | 0 | 0 | 0 | 0 |
| Bałcyny | Tamadur | 40 | 1.2 | 0 | 0 | 0 | 0 |
| Bałcyny | Floradur | 40 | 1.2 | 0 | 0 | 0 | 0 |
| Bałcyny | Floradur | 29 | 1.6 | 5.8 | 1 | 0 | 0 |
| Bałcyny | Duralis | 40 | 1.33 | 4 | 1 | 0 | 0 |
| Bałcyny | Duralis | 48 | 1.3 | 0 | 0 | 0 | 0 |
| Bałcyny | Duramant | 84 | 1.5 | 4 | 1 | 0 | 0 |
| Bałcyny | Duramant | 72 | 1.4 | 0 | 0 | 0 | 0 |
| Bałcyny | Duranegra | 56 | 1.57 | 0 | 0 | 0 | 0 |
| Bałcyny | Duranegra | 68 | 1.35 | 0 | 0 | 0 | 0 |
| Bałcyny | Durasol | 28 | 1 | 4 | 2 | 0 | 0 |
| Bałcyny | Durasol | 37 | 1.28 | 0 | 0 | 0 | 0 |
| NK | Duragold | 31.25 | 1.6 | 14.6 | 1 | 0 | 0 |
| NK | Duralis | 65.31 | 1.5 | 4.01 | 2 | 0 | 0 |
| NK | Floradur | 69.04 | 1.21 | 9.52 | 1 | 0 | 0 |
| NK | Duramant | 38.23 | 1.61 | 2.91 | 1 | 0 | 0 |
| NK | Tamadur | 60 | 1.28 | 6.67 | 1 | 0 | 0 |
| NK | Duranegra | 43.3 | 1.15 | 10 | 1 | 0 | 0 |
