# Supplementary material for: Screening durum wheat cultivars for resistance traits against the stem-base pathogen Fusarium graminearum
Source: PeerJ. 2025 Nov 13;13:e20105. doi: 10.7717/peerj.20105 (PMC12619947; doi:10.7717/peerj.20105)
Supplement: Supplementary file 9 [file peerj-13-20105-s009.odt]

| Cultivar | Percentage of healthy seedlings | Percentage of infected seedlings | Percentage of ungerminated kernels | Infection severity on seedlings* | Seedling height (cm) | Root length (cm) |
| --- | --- | --- | --- | --- | --- | --- |
| Tamadur | 40 | 15 | 45 | 1 | 22.5 | 20.5 |
| Tamadur | 40 | 20 | 40 | 2.2 | 18.3 | 18.4 |
| Tamadur | 15 | 30 | 55 | 3 | 21 | 18 |
| Tamadur | 30 | 35 | 35 | 2.7 | 15.3 | 20.3 |
| Floradur | 40 | 15 | 45 | 1 | 24 | 21 |
| Floradur | 45 | 15 | 40 | 1.6 | 18 | 21 |
| Floradur | 15 | 20 | 65 | 2.3 | 50 | 21 |
| Floradur | 10 | 15 | 75 | 3 | 19.5 | 21 |
| Duragold | 20 | 15 | 65 | 1.25 | 21.8 | 21 |
| Duragold | 25 | 20 | 55 | 1.25 | 16.3 | 14.5 |
| Duragold | 40 | 20 | 40 | 2 | 20 | 20.8 |
| Duragold | 10 | 35 | 55 | 2.3 | 13.3 | 17 |
| Duranegra | 15 | 40 | 45 | 1.6 | 20.3 | 22.3 |
| Duranegra | 15 | 25 | 60 | 2 | 19 | 20 |
| Duranegra | 5 | 30 | 15 | 3 | 20 | 20 |
| Duranegra | 15 | 40 | 45 | 3 | 14 | 20 |
| Durasol | 20 | 30 | 50 | 3 | 17.8 | 19.3 |
| Durasol | 25 | 35 | 40 | 2 | 16.5 | 19.8 |
| Durasol | 45 | 25 | 30 | 1.7 | 20.6 | 20 |
| Durasol | 35 | 25 | 40 | 2 | 18 | 20.5 |
| Tamadur | 35 | 25 | 40 | 0.7 | 17.8 | 24.8 |
| Tamadur | 0 | 40 | 60 |  |  |  |
| Tamadur | 10 | 35 | 55 | 1 | 16 | 24 |
| Tamadur | 15 | 40 | 45 |  | 16.3 | 14.3 |
| Floradur | 45 | 15 | 40 | 2 | 17.5 | 21.5 |
| Floradur | 40 | 15 | 45 | 1.6 | 22 | 21.5 |
| Floradur | 45 | 35 | 20 | 2 | 21.5 | 22.5 |
| Floradur | 30 | 20 | 50 | 1.8 | 21 | 25 |
| Duragold | 55 | 10 | 35 | 1.5 | 17.8 | 24 |
| Duragold | 15 | 30 | 55 | 2 | 15 | 20 |
| Duragold | 20 | 25 | 55 | 2.5 | 17.5 | 21 |
| Duragold | 15 | 25 | 60 | 3 | 18 | 21 |
| Duranegra | 0 | 45 | 55 |  |  |  |
| Duranegra | 25 | 30 | 45 | 2.4 | 19.8 | 22 |
| Duranegra | 35 | 30 | 35 | 2 | 16.9 | 22.8 |
| Duranegra | 15 | 50 | 35 | 1.7 | 15 | 21 |
| Durasol | 20 | 30 | 50 | 1.3 | 19.5 | 21 |
| Durasol | 15 | 30 | 55 | 3 | 19.3 | 20.7 |
| Durasol | 20 | 35 | 45 | 2.3 | 18.3 | 19.2 |
| Durasol | 10 | 35 | 55 | 1 | 17 | 18.5 |
| Tamadur | 55 | 15 | 30 | 0 | 15 | 24.4 |
| Tamadur | 40 | 15 | 45 | 0.8 | 14.2 | 24.4 |
| Tamadur | 5 | 70 | 25 | 3 | 9 | 20 |
| Tamadur | 30 | 45 | 25 | 2.4 | 16.8 | 23.3 |
| Floradur | 10 | 65 | 25 | 1.5 | 6.5 | 16 |
| Floradur | 55 | 5 | 40 | 1.6 | 13.8 | 23 |
| Floradur | 40 | 40 | 20 | 0.8 | 14.2 | 23.2 |
| Floradur | 20 | 0 | 80 | 0.25 | 10.5 | 22.75 |
| Duragold | 65 | 25 | 10 | 1.6 | 13.2 | 23 |
| Duragold | 40 | 40 | 20 | 1.8 | 13 | 24 |
| Duragold | 70 | 20 | 40 | 1 | 13.2 | 20 |
| Duragold | 50 | 45 | 5 | 1.7 | 14.4 | 24 |
| Duranegra | 50 | 45 | 5 | 1.9 | 14.2 | 23.8 |
| Duranegra | 20 | 65 | 20 | 3 | 13.2 | 24 |
| Duranegra | 25 | 50 | 25 | 2.2 | 12.8 | 24.4 |
| Duranegra | 15 | 75 | 10 | 2.3 | 13.3 | 24 |
| Durasol | 40 | 30 | 30 | 2 | 13.8 | 23.8 |
| Durasol | 30 | 25 | 45 | 1.3 | 14.4 | 23.4 |
| Durasol | 15 | 50 | 35 | 2.7 | 11 | 23 |
| Durasol | 25 | 70 | 5 | 2.2 | 12.4 | 23.6 |
