# Supplementary material for: Screening durum wheat cultivars for resistance traits against the stem-base pathogen Fusarium graminearum
Source: PeerJ. 2025 Nov 13;13:e20105. doi: 10.7717/peerj.20105 (PMC12619947; doi:10.7717/peerj.20105)
Supplement: Supplementary file 10 [file peerj-13-20105-s010.odt]

| Treatment | Cultivar | NDVI |
| --- | --- | --- |
| Control | Tamadur | 0.521 |
| Control | Tamadur | 0.5683 |
| Control | Tamadur | 0.505 |
| Control | Tamadur | 0.399 |
| Control | Tamadur | 0.4751 |
| Control | Tamadur | 0.5166 |
| Control | Tamadur | 0.5156 |
| Control | Tamadur | 0.4665 |
| Control | Floradur | 0.4498 |
| Control | Floradur | 0.5847 |
| Control | Floradur | 0.4507 |
| Control | Floradur | 0.5658 |
| Control | Floradur | 0.3875 |
| Control | Floradur | 0.3875 |
| Control | Floradur | 0.5416 |
| Control | Floradur | 0.4914 |
| Control | Duragold | 0.5063 |
| Control | Duragold | 0.3796 |
| Control | Duragold | 0.5972 |
| Control | Duragold | 0.6103 |
| Control | Duragold | 0.5168 |
| Control | Duragold | 0.6086 |
| Control | Duragold | 0.5261 |
| Control | Duragold | 0.5581 |
| Control | Duranegra | 0.5685 |
| Control | Duranegra | 0.5045 |
| Control | Duranegra | 0.6137 |
| Control | Duranegra | 0.6164 |
| Control | Duranegra | 0.5702 |
| Control | Duranegra | 0.6122 |
| Control | Duranegra | 0.5904 |
| Control | Duranegra | 0.5860 |
| Control | Durasol | 0.5727 |
| Control | Durasol | 0.4886 |
| Control | Durasol | 0.5490 |
| Control | Durasol | 0.5312 |
| Control | Durasol | 0.4558 |
| Control | Durasol | 0.5405 |
| Control | Durasol | 0.4746 |
| Control | Durasol | 0.5113 |
| Control | Tamadur | 0.5155 |
| Control | Tamadur | 0.5276 |
| Control | Tamadur | 0.4448 |
| Control | Tamadur | 0.2992 |
| Control | Tamadur | 0.5401 |
| Control | Tamadur | 0.5504 |
| Control | Tamadur | 0.4814 |
| Control | Tamadur | 0.4443 |
| Control | Floradur | 0.5151 |
| Control | Floradur | 0.5349 |
| Control | Floradur | 0.4897 |
| Control | Floradur | 0.5504 |
| Control | Floradur | 0.591 |
| Control | Floradur | 0.5447 |
| Control | Floradur | 0.5272 |
| Control | Floradur | 0.4918 |
| Control | Duragold | 0.5548 |
| Control | Duragold | 0.5969 |
| Control | Duragold | 0.5686 |
| Control | Duragold | 0.5553 |
| Control | Duragold | 0.5239 |
| Control | Duragold | 0.6291 |
| Control | Duragold | 0.5301 |
| Control | Duragold | 0.6001 |
| Control | Duranegra | 0.4684 |
| Control | Duranegra | 0.5700 |
| Control | Duranegra | 0.5456 |
| Control | Duranegra | 0.5573 |
| Control | Duranegra | 0.6010 |
| Control | Duranegra | 0.5715 |
| Control | Duranegra | 0.5349 |
| Control | Duranegra | 0.5168 |
| Control | Durasol | 0.4567 |
| Control | Durasol | 0.4428 |
| Control | Durasol | 0.3671 |
| Control | Durasol | 0.6217 |
| Control | Durasol | 0.5574 |
| Control | Durasol | 0.5938 |
| Control | Durasol | 0.5132 |
| Control | Durasol | 0.6003 |
| Fung | Tamadur | 0.543 |
| Fung | Tamadur | 0.2797 |
| Fung | Tamadur | 0.4655 |
| Fung | Tamadur | 0.3839 |
| Fung | Tamadur | 0.4424 |
| Fung | Tamadur | 0.4152 |
| Fung | Tamadur | 0.4703 |
| Fung | Tamadur | 0.4633 |
| Fung | Floradur | 0.4163 |
| Fung | Floradur | 0.5208 |
| Fung | Floradur | 0.3623 |
| Fung | Floradur | 0.4436 |
| Fung | Floradur | 0.3875 |
| Fung | Floradur | 0.506 |
| Fung | Floradur | 0.3981 |
| Fung | Floradur | 0.5522 |
| Fung | Duragold | 0.3626 |
| Fung | Duragold | 0.4286 |
| Fung | Duragold | 0.5876 |
| Fung | Duragold | 0.5763 |
| Fung | Duragold | 0.4925 |
| Fung | Duragold | 0.4744 |
| Fung | Duragold | 0.4906 |
| Fung | Duragold | 0.5006 |
| Fung | Duranegra | 0.5172 |
| Fung | Duranegra | 0.4545 |
| Fung | Duranegra | 0.4794 |
| Fung | Duranegra | 0.5516 |
| Fung | Duranegra | 0.5567 |
| Fung | Duranegra | 0.4371 |
| Fung | Duranegra | 0.5344 |
| Fung | Duranegra | 0.5093 |
| Fung | Durasol | 0.4553 |
| Fung | Durasol | 0.4462 |
| Fung | Durasol | 0.3024 |
| Fung | Durasol | 0.5487 |
| Fung | Durasol | 0.3085 |
| Fung | Durasol | 0.5619 |
| Fung | Durasol | 0.3001 |
| Fung | Durasol | 0.395 |
| Fung | Tamadur | 0.5525 |
| Fung | Tamadur | 0.5958 |
| Fung | Tamadur | 0.5756 |
| Fung | Tamadur | 0.4672 |
| Fung | Tamadur | 0.1408 |
| Fung | Tamadur | 0.2299 |
| Fung | Tamadur | 0.5367 |
| Fung | Tamadur | 0.4053 |
| Fung | Floradur | 0.5329 |
| Fung | Floradur | 0.4324 |
| Fung | Floradur | 0.4505 |
| Fung | Floradur | 0.461 |
| Fung | Floradur | 0.3611 |
| Fung | Floradur | 0.4779 |
| Fung | Floradur | 0.4237 |
| Fung | Floradur | 0.5111 |
| Fung | Duragold | 0.5397 |
| Fung | Duragold | 0.3617 |
| Fung | Duragold | 0.3606 |
| Fung | Duragold | 0.4981 |
| Fung | Duragold | 0.3984 |
| Fung | Duragold | 0.4445 |
| Fung | Duragold | 0.4355 |
| Fung | Duragold | 0.525 |
| Fung | Duranegra | 0.4452 |
| Fung | Duranegra | 0.4162 |
| Fung | Duranegra | 0.4203 |
| Fung | Duranegra | 0.4461 |
| Fung | Duranegra | 0.5431 |
| Fung | Duranegra | 0.5187 |
| Fung | Duranegra | 0.3593 |
| Fung | Duranegra | 0.5284 |
| Fung | Durasol | 0.5003 |
| Fung | Durasol | 0.5851 |
| Fung | Durasol | 0.5171 |
| Fung | Durasol | 0.5718 |
| Fung | Durasol | 0.4409 |
| Fung | Durasol | 0.4742 |
| Fung | Durasol | 0.3524 |
| Fung | Durasol | 0.5596 |
| Biol | Tamadur | 0.5316 |
| Biol | Tamadur | 0.5241 |
| Biol | Tamadur | 0.4548 |
| Biol | Tamadur | 0.5243 |
| Biol | Tamadur | 0.4723 |
| Biol | Tamadur | 0.4612 |
| Biol | Tamadur | 0.5753 |
| Biol | Tamadur | 0.6208 |
| Biol | Floradur | 0.5282 |
| Biol | Floradur | 0.5233 |
| Biol | Floradur | 0.5563 |
| Biol | Floradur | 0.4928 |
| Biol | Floradur | 0.5943 |
| Biol | Floradur | 0.5013 |
| Biol | Floradur | 0.5782 |
| Biol | Floradur | 0.6153 |
| Biol | Duragold | 0.6025 |
| Biol | Duragold | 0.5387 |
| Biol | Duragold | 0.5596 |
| Biol | Duragold | 0.6142 |
| Biol | Duragold | 0.478 |
| Biol | Duragold | 0.6338 |
| Biol | Duragold | 0.5625 |
| Biol | Duragold | 0.5637 |
| Biol | Duranegra | 0.5791 |
| Biol | Duranegra | 0.6401 |
| Biol | Duranegra | 0.5188 |
| Biol | Duranegra | 0.5966 |
| Biol | Duranegra | 0.5015 |
| Biol | Duranegra | 0.5915 |
| Biol | Duranegra | 0.5953 |
| Biol | Duranegra | 0.6420 |
| Biol | Durasol | 0.5509 |
| Biol | Durasol | 0.5346 |
| Biol | Durasol | 0.5052 |
| Biol | Durasol | 0.5895 |
| Biol | Durasol | 0.5826 |
| Biol | Durasol | 0.606 |
| Biol | Durasol | 0.4910 |
| Biol | Durasol | 0.5954 |
| Biol | Tamadur | 0.5532 |
| Biol | Tamadur | 0.5727 |
| Biol | Tamadur | 0.4548 |
| Biol | Tamadur | 0.5382 |
| Biol | Tamadur | 0.555 |
| Biol | Tamadur | 0.4884 |
| Biol | Tamadur | 0.5225 |
| Biol | Tamadur | 0.5385 |
| Biol | Floradur | 0.6103 |
| Biol | Floradur | 0.6222 |
| Biol | Floradur | 0.5722 |
| Biol | Floradur | 0.5864 |
| Biol | Floradur | 0.5743 |
| Biol | Floradur | 0.6097 |
| Biol | Floradur | 0.5441 |
| Biol | Floradur | 0.5162 |
| Biol | Duragold | 0.5919 |
| Biol | Duragold | 0.5848 |
| Biol | Duragold | 0.5944 |
| Biol | Duragold | 0.6216 |
| Biol | Duragold | 0.5674 |
| Biol | Duragold | 0.5919 |
| Biol | Duragold | 0.5679 |
| Biol | Duragold | 0.5852 |
| Biol | Duranegra | 0.5265 |
| Biol | Duranegra | 0.6059 |
| Biol | Duranegra | 0.5996 |
| Biol | Duranegra | 0.5850 |
| Biol | Duranegra | 0.6474 |
| Biol | Duranegra | 0.5815 |
| Biol | Duranegra | 0.6343 |
| Biol | Duranegra | 0.6092 |
| Biol | Durasol | 0.4767 |
| Biol | Durasol | 0.5863 |
| Biol | Durasol | 0.4841 |
| Biol | Durasol | 0.5293 |
| Biol | Durasol | 0.5555 |
| Biol | Durasol | 0.6113 |
| Biol | Durasol | 0.4998 |
| Biol | Durasol | 0.4825 |
